# NAT10-Mediated ac4C-Modification Exacerbates Ferroptosis by Stabilizing HMOX1 in Deep Vein Thrombosis

**DOI:** 10.1161/ATVBAHA.125.323986

**Published:** 2025-12-30

**Authors:** Yunhong Zhang, Zhen Zhang, Xinkui Liu, Chu Chu, Xiaoyan Yu, Qiaoqiao Han, Wei Li, Tingting Zhang, Huiyan Zu, Nannan Fan, Ran Wei, Feifei Shi, Fang Li, Fei Xu, Bin Wang, Xia Li

**Affiliations:** 1 Innovative Institute of Chinese Medicine and Pharmacy, Shandong University of Traditional Chinese Medicine, Jinan, China (Y.Z., Z.Z., X. Liu, C.C., W.L., T.Z., H.Z., N.F., F.S., F.L., X. Li).; 2 Key Laboratory of Traditional Chinese Medicine Classical Theory, Ministry of Education, Jinan Key Laboratory of Traditional Chinese Medicine Immunoregulation, Traditional Chinese Medicine Immunoregulation Engineering Research Center of Shandong Province, Key Laboratory of Interdisciplinary Innovation for Traditional Chinese Medicine and Immunology, Shandong University of Traditional Chinese Medicine, Jinan, China (Y.Z., Z.Z., X. Liu, C.C., W.L., T.Z., H.Z., N.F., F.S., F.L., X. Li).; 3 College of Traditional Chinese Medicine, Shandong University of Traditional Chinese Medicine, Jinan, China (X. Li).; 4 School of Clinical and Basic Medical Sciences, Shandong First Medical University and Shandong Academy of Medical Sciences, Jinan, China (R.W.).; 5 Department of Respiratory and Critical Care Medicine, Affiliated Hospital of Shandong University of Traditional Chinese Medicine, Jinan (F.X.).; 6 The Second Affiliated Hospital of Shandong University of Traditional Chinese Medicine, Jinan (B.W.).

**Keywords:** deep vein thrombosis, endothelial cells, ferroptosis, *N*4-acetylcytidine, *N*-acetyltransferase 10

## Abstract

**BACKGROUND:**

Deep vein thrombosis (DVT) is a prevalent peripheral vascular disorder associated with abnormal epigenetic processes and altered gene expression in endothelial cells. Accumulating evidence has demonstrated that NAT10 (*N*-acetyltransferase 10)–mediated *N*4-acetylcytidine modification exerts unique roles in ferroptosis, but its roles are still elusive in DVT.

**METHODS:**

To explore the potential mechanism of NAT10 and ferroptosis on thrombogenesis, we used NAT10 and GPX4 (glutathione peroxidase 4) knockout mice as an in vivo model, and utilized techniques, such as RNA immunoprecipitation, acRIP-qPCR (acetylated RNA immunoprecipitation-quantitative PCR), *N*4-acetylcytidine Dot Blotting assay, and Western blotting, for detailed molecular analysis.

**RESULTS:**

GPX4 is a pivotal gene that suppresses ferroptosis. Utilizing endothelial cell–specific GPX4 conditional knockout mice (GPX4^fl/fl^Cdh5-Cre^+^), we proved that ferroptosis in endothelial cells promotes the formation of thrombosis. Previous evidence indicates that NAT10 overexpression induces ferroptosis and downregulates GPX4 expression. Here, we found that NAT10 expression was elevated in DVT mice, and silencing of NAT10 markedly attenuated ferroptosis both in vitro and in vivo. Furthermore, endothelial cell–specific knockout of NAT10 (NAT10^fl/fl^Cdh5-Cre^+^) demonstrated a reduction in endothelial ferroptosis, thereby inhibiting both the formation and progression of DVT. Mechanistic studies indicated that NAT10 facilitated the N4-acetylcytidine modification of HMOX1 (heme oxygenase 1), which enhanced its mRNA stability, leading to the accumulation of ferrous ions, and exacerbating endothelial dysfunction in DVT.

**CONCLUSIONS:**

Collectively, our data elucidate that downregulation of NAT10 mitigates endothelial ferroptosis and prevents DVT formation and progression by modulating HMOX1 expression, which offers a potential novel strategy for the prevention and treatment of thrombosis in DVT.

**What Are the Clinical Implications?**

This study reveals that NAT10 (*N*-acetyltransferase 10)–mediated N4-acetylcytidine RNA modification serves as a critical epigenetic mechanism driving endothelial ferroptosis and exacerbating deep vein thrombosis pathogenesis. Quantitative proteomic profiling of experimental murine deep vein thrombosis models revealed an elevation in NAT10 expression. Endothelial-specific deletion of NAT10 or pharmacological inhibition via remodelin markedly reduced thrombus burden and attenuated disease progression across independent in vivo models. Mechanistically, NAT10 catalyzes *N*4-acetylcytidine modification of HMOX1 (heme oxygenase 1) mRNA, thereby enhancing its stability and ultimately resulting in the accumulation of HMOX1 protein. This molecular cascade promotes ferroptotic endothelial cell death via iron-dependent lipid peroxidation, as evidenced by reduced glutathione and elevated malondialdehyde. Conversely, endothelial-specific restoration of HMOX1 expression rescued the NAT10-loss–mediated protective phenotype, establishing HMOX1 as a requisite downstream effector. Collectively, our findings identify the NAT10-HMOX1 regulatory axis as a previously unrecognized driver of deep vein thrombosis pathobiology and posit pharmacological NAT10 inhibition as a translational strategy to blunt ferroptosis and prevent thrombosis.

Deep vein thrombosis (DVT) is a common peripheral vascular condition characterized by an estimated annual incidence of >1 case per 1000 individuals.^1^ The rising incidence of DVT has a profound impact on patients’ health and overall quality life.^2,3^ The primary etiological factors contributing to DVT include endothelial damage, venous stasis, and hypercoagulability.^4^ A thorough comprehension of the underlying mechanisms of endothelial injury is essential for elucidating the initiation of DVT.^5,6^ Despite significant efforts aimed at investigating the associated molecular mechanisms, there remains a notable lack of viable therapeutic targets for clinically mitigating vascular injury.

Emerging evidence suggests a significant association between elevated iron levels and thrombosis, underscoring the pivotal role of iron homeostasis in thrombotic events.^7,8^ Recent studies have identified ferroptosis, a regulated form characterized by iron overload and lipid peroxidation, as being linked to vascular injury.^9,10^ Iron accumulation significantly accelerates thrombosis after vascular injury and further enhances vascular oxidative stress.^11^ In addition, the presence of excessive free divalent iron exacerbates oxidative stress within cells, thereby promoting ferroptosis.^12^ Although iron homeostasis and ferroptosis are associated with endothelial injury and thrombosis, the precise regulatory mechanisms linking iron homeostasis and ferroptosis in DVT formation remain unclear.

N4-acetylcytidine (ac4C), a recently identified mRNA modification, is recognized as a prevalent epigenetic marker of mRNAs that plays a critical role in regulating mRNA stability or translation efficiency.^13–15^ NAT10 (*N*-acetyltransferase 10) is the only known ac4C writer in mammals, catalyzing the acetylation of cytidine in mRNAs.^16^ In addition, NAT10 regulates ribosome biogenesis and cytokinesis.^17^ Emerging studies have linked NAT10-mediated ac4C modification to various physiological and pathological processes, including cellular senescence, apoptosis, autophagy, and ferroptosis.^18–21^ Nevertheless, the precise alterations and functional significance of NAT10 in ferroptosis associated with DVT have yet to be thoroughly elucidated.

HMOX1 (heme oxygenase 1), an activator of ferroptosis, can decompose heme into carbon monoxide, biliverdin, and ferrous ions (Fe^2+^), which promote the accumulation of lipid peroxidation by producing reactive oxygen species through the Fenton reaction.^22,23^ Although previous studies have suggested that HMOX1 serves a cytoprotective function, accumulating evidence indicates that its cytotoxic effects emerge when its intracellular levels exceed a certain threshold.^24,25^ More importantly, excessive expression of HMOX1 induces Fe^2+^ overload, enhances ROS production, aggravates lipid peroxidation, and ultimately facilitates ferroptosis in endothelial cells.^26^ However, the involvement of HMOX1 in the pathogenesis of DVT has not been sufficiently characterized.

In this study, we demonstrate that NAT10 serves as a notable promoter of endothelial ferroptosis, with its expression dramatically elevated in DVT. Our findings uncovered that inhibition of NAT10 attenuated endothelial ferroptosis by reducing ac4C levels in DVT. Mechanistically, the reduction of NAT10 leads to diminished stabilization of HMOX1, which subsequently results in decreased release of Fe^2+^ and lower levels of lipid peroxides. Taken together, our findings suggest that targeting the NAT10/HMOX1 axis may emerge as a promising therapeutic strategy for the treatment of DVT.

## Materials and Methods

### Data Availability

The acetylated RNA immunoprecipitation (RIP) sequencing (acRIP-seq) data (GSE307103) used in this study are publicly accessible through the GEO database, and the proteomics data have been deposited to the ProteomeXchange Consortium with the data set identifier of PXD067933. The data that support the findings of this study are available from the corresponding authors on reasonable request.

### Microarray Analysis

The genome-wide analysis of mRNA expression of peripheral blood mononuclear cells in DVT patients (n=6) and control subjects (n=6) was performed using Human mRNA (4*180K, Design ID: 084410, Table S1). This was performed by OE Biotech, Co, Ltd (Shanghai, China) in accordance with the standard protocols. This study was approved by the ethics committee of Affiliated Hospital of Shandong University of Traditional Chinese Medicine, and written informed consent was obtained from all participants (approval number: [2019] Lun Shen No. [008]-KY).

### Proteomics

Vascular tissues were collected for protein extraction by centrifugation in DVT mice (with thrombus, n=6), DVT mice (without thrombus, n=6), and control subjects (n=6). Then, 4-dimensional (4D) label-free quantitation proteomics analysis, including liquid chromatography–MS/MS analysis, and data analysis were performed. The proteomics analysis was supported by Jingjie PTM BioLabs (Hangzhou, China).

### Cell Culture and Treatment

Human umbilical vein endothelial cells (HUVECs) and C166 cells were obtained from BeNa Culture Collection (Hebei, China). Cells were transfected with siRNAs (Table S2) or a negative control (GenePharma, Shanghai, China) by Lipofectamine RNAiMAX (Invitrogen, Carlsbad) according to the manufacturer’s instructions. The GV314 (CMV-MCS-3FLAG-SV40-EGFP) adenoviral system (GeneChem, Shanghai, China) was used to obtain the NAT10 overexpression adenovirus (Ad-NAT10) and the negative control adenovirus. HUVECs were cultured at a density of 1×10^5^ in a 12-well culture plate for 12 hours, then adenovirus (MOI=200) was added to the culture medium for subsequent experiments. In addition, endothelial dysfunction was induced by treatment with 100 μM Ang II (angiotensin II) in HUVECs.^27^

### Determination of Iron Content

The relative Fe^2+^ content in the vascular tissues was analyzed using a ferrous ion content assay kit (BC5415; Solarbio, Beijing, China). The absorbance was gauged at 593 nm using a microplate reader (SpectraMax i3x; Molecular Devices, Shanghai, China).

### Measurement of Malondialdehyde Levels

The treated cells and venous vessels were lysed via sonication, and all lysis steps were carried out in an ice bath. The relative malondialdehyde levels were measured using the Malondialdehyde Content Assay Kit (BC0025; Solarbio, Beijing, China).

### Glutathione Measurement

The treated cells and venous vessels were lysed by sonication and homogenized in an ice bath. Reduced glutathione Content Assay Kit (BC1175; Solarbio, Beijing, China) was used to assay the relative glutathione level.

### Measurements of Intracellular Lipid Peroxidation

Pretreated cells were incubated with the BODIPYTM 581/591 C11 probe (Invitrogen, Carlsbad) for 30 minutes at 37 °C. Cells were plated on confocal culture dishes and treated with RSL3 or FINO2 (ferroptosis activator) in the absence or presence of Remodelin for 12 hours. After 3 washes with PBS, the nucleus was stained with Hoechst (Beyotime, Shanghai, China). Lipid peroxidation fluorescence was measured by using a scanning confocal microscope (LSM 880; Carl Zeiss AG, Oberkochen, Germany).

### Immunofluorescence for Fe^2+^ Analysis

FerroOrange (Dojindo Laboratories, Kumamoto, Japan) was used to determine Fe^2+^ levels in cells treated with different reagents. Ultimately, a scanning confocal microscope was used to capture the images.

### Cell Viability Assays

Cells were seeded in 96-well plates at 8×10^3^ cells per well. After the experimental protocol, the culture medium was changed into 100 μL DMEM and 10 μL CCK8 solution (Beyotime, Shanghai, China) per well. After incubation at 37 °C for 1 hour, absorbance was measured at 450 nm.

### Quantitative Real-Time Polymerase Chain Reaction

Total RNA was extracted with TRIzol Reagent (CWBIO, Beijing, China) after specification. Roughly 1 µg of RNA was converted to cDNA, which was performed using SYBR Green (CWBIO, Beijing, China), and the relative mRNA levels were calculated using the 2^−ΔΔCt^ formula. The polymerase chain reaction (PCR) primer sequences are shown in Table S3.

### RNA Immunoprecipitation

The RIP kit (Geneseed Biotech, Guangzhou, China) was used to capture the antigen after the magnetic beads were connected to the NAT10 antibody or IgG antibody. RNAs were then extracted, and quantitative real-time PCR (qRT-PCR) was used for verification.

### Acetylated RIP Sequencing

HUVECs were transfected and subjected to acRIP-seq by Cloud-Seq Inc (Shanghai, China). The RNA is first extracted from the sample (extracted using TRIzol), the RNA is quality controlled, and the concentration and purity of the RNA are detected. Total RNA was subjected to immunoprecipitation according to the manufacturer’s instructions. The samples were sequenced using a NovaSeq platform (Illumina).

### acRIP-qPCR

The ac4C RIP assay was performed using GenSeq ac4C RIP Kit (GS-ET-005; Cloud-Seq Biotech, Shanghai, China) following the manufacturer’s instructions. Then, the RNAs were purified, and the enrichment of HMOX1 mRNA was analyzed by qRT-PCR. Primers were listed in Table S4.

### Western Blotting

Samples were lysed in RIPA buffer with protease and phosphatase inhibitors (Solarbio, Beijing, China), and protein concentration was determined using the BCA Protein Assay Kit (Beyotime, Shanghai, China). Protein samples were denatured, separated by SDS-PAGE, and transferred for immunoblotting with antibodies. Following transfer, the membranes were further cut into smaller sections to reduce antibody consumption and fit the incubation plates. Antibodies against NAT10 (1:1000, ab194297; Abcam, Cambridge, United Kingdom), HMOX1 (1:1000, ab68477; Abcam, Cambridge, United Kingdom), GPX4 (glutathione peroxidase 4; 1:1000, ab125066; Abcam, Cambridge, United Kingdom), and GAPDH (1:10 000, ab181603; Abcam, Cambridge, United Kingdom) were used in this study.

### RNA ac4C Dot Blotting assay

Total RNAs from venous vessels were extracted and denatured at 95 °C for 3 minutes. Subsequently, RNA samples were spotted on a nylon membrane, which was cross-linked for 30 minutes at 37 °C. The membrane was blocked with 5% skim milk and incubated with anti-ac4C antibody (1:500, ab252215; Abcam, Cambridge) overnight at 4 °C. After incubating with horseradish peroxidase–conjugated anti-rabbit IgG secondary antibody (1:4000, ZSGB-Bio, Beijing, China), the membrane was visualized by Tanon 5200 Multichemiluminescent Imaging System (Tanon, Shanghai, China).

### RNA Stability Assay

HUVECs were transfected with NC or si-NAT10s, followed by treatment with actinomycin D (AbMole, Houston) at a final concentration of 5 μg/mL. After 0, 2, 4, or 6 hours of incubation, cells were collected, and RNAs were isolated for qRT-PCR.

### Construction and Generation of Endothelial-Specific NAT10 Knockout Mice

Endothelial-specific NAT10 knockout mice were purchased from Cyagen Biosciences (Suzhou, China). Endothelium-specific conditional NAT10 deficiency (C57BL/6J, NAT10^flox/flox^Cdh5-Cre^ERT2^) mice were generated by crossing NAT10^flox/flox^ mice with Cdh5-Cre^ERT2^ mice. NAT10^fl/fl^Cdh5-Cre^+^ (NAT10^flox/flox^Cdh5-Cre^ERT2^) and NAT10^fl/fl^Cdh5-Cre^−^ (NAT10^flox/flox^) groups received identical tamoxifen treatment via intraperitoneal injection with 60 mg/kg daily for 7 consecutive days.^28^ The genotypes of NAT10 knockout mice were confirmed by PCR. Western blotting and qRT-PCR were used to identify the knockout efficiency of NAT10^fl/fl^Cdh5-Cre^+^ mice. Primers for NAT10 and Cdh5-Cre^ERT2^ transgene are in Table S5.

### Construction and Generation of Endothelial-Specific GPX4 Knockout Mice

Endothelial-specific GPX4 knockout mice were obtained from Shanghai Jiao Tong University. Endothelium-specific conditional GPX4 deficiency (C57BL/6J, GPX4^flox/flox^Cdh5-Cre^ERT2^) mice were generated by crossing GPX4^flox/flox^ mice with Cdh5-Cre^ERT2^ mice. GPX4^fl/fl^Cdh5-Cre^+^ (GPX4^flox/flox^Cdh5-Cre^ERT2^) and GPX4^fl/fl^Cdh5-Cre^-^ (GPX4^flox/flox^) groups received identical tamoxifen treatment via intraperitoneal injection with 60 mg/kg daily for 7 consecutive days.^28^ Western blotting and qRT-PCR were used to identify the knockout efficiency of GPX4^fl/fl^Cdh5-Cre^+^ mice. Primers for GPX4 and Cdh5-Cre^ERT2^ transgene are in Table S5.

### DVT Mice Model and Treatment

Wild-type mice (C57BL/6J, 8-week-old male) were purchased from Beijing HFK Bioscience Company (Beijing, China). The mice were fed a standard SPF maintenance diet (catalog no. 23103213) from Beijing Xieli Feed Co, Ltd, consisting of corn, soybean meal, fish meal, wheat flour, yeast powder, vegetable oil, salt, vitamins, and mineral elements. It has been demonstrated that estrogen can promote thrombosis.^29,30^ Therefore, we used male mice in this study to avoid the possible effects of estrogen. Animal experiments were approved by the Institutional Animal Care and Use Committee of Shandong University of Traditional Chinese Medicine. The model of DVT mice was established in accordance with the inferior vena cava (IVC) stenosis method.^31^ In another DVT model, the IVC was exposed, and a piece of filter paper soaked in 10% ferric-chloride (FeCl_3_) solution was placed on the IVC for 3 minutes.^32^ Remodelin (10 mg/kg) was intraperitoneally injected for 3 consecutive days before FeCl_3_ induction and continued for 4 consecutive days after surgery. Endothelial cells isolated from mouse vascular tissues using the Mouse Vascular Endothelial Cell Isolation Kit (VE2011M, TBDsciences, Tianjin, China).

For ferroptosis inhibitor experiments, mice were stochastically split into 3 groups (20 per group): (1) sham group; (2) DVT group; (3) DVT+Fer-1 (ferrostatin-1, ferroptosis inhibitor, Aladdin, China) group. Fer-1 (5 mg/kg) was injected intraperitoneally for 3 consecutive days before the mice underwent IVC stenosis and continued for 4 consecutive days after surgery.^33^

For HMOX1 inhibitor experiments, mice were randomly assigned to 2 groups (20/group): (1) DVT group; (2) DVT+ZnPP (zinc protoporphyrin IX, HMOX1 inhibitor, Aladdin, China) group. ZnPP (10 mg/kg) was injected intraperitoneal injection which was initiated 3 days before the mice underwent IVC stenosis and continued for 4 consecutive days after surgery.^34^

For NAT10 treatment experiments, mice were stochastically split into 3 groups (20 per group): (1) sham group; (2) DVT group; (3) DVT+remodelin (NAT10 inhibitor, Aladdin, China) group. Remodelin (10 mg/kg) was intraperitoneally injected for 3 consecutive days before the mice underwent IVC stenosis and continued for 4 consecutive days after surgery.

For HMOX1 overexpression experiments, mice were stochastically split into 3 groups (20/group): (1) DVT group (NAT10^fl/fl^Cdh5-Cre^−^ mice); (2) DVT NAT10-knockout group (NAT10^fl/fl^Cdh5-Cre^+^ mice); (3) DVT NAT10-knockout+CoPP (cobalt protoporphyrin IX; HMOX1-inducer cobalt protoporphyrin; Aladdin, China) group (NAT10^fl/fl^Cdh5-Cre^+^+CoPP mice). CoPP (15 mg/kg) was injected intraperitoneally for 3 consecutive days before the mice underwent IVC stenosis and continued for 4 consecutive days after surgery.^35^

For HMOX1 overexpression experiments, mice were stochastically split into 3 groups (20 per group): (1) DVT group (NAT10^fl/fl^Cdh5-Cre^-^ mice); (2) DVT NAT10-knockout+adeno-associated virus (AAV)–EV (control virus) group (NAT10^fl/fl^Cdh5-Cre^+^+AAV-EV mice); (3) DVT NAT10-knockout+AAV-HMOX1 (HMOX1 overexpression; GeneChem, Shanghai, China) group (NAT10^fl/fl^Cdh5-Cre^+^+AAV-HMOX1 mice). Then, 100 µL (5×10^12^ v.g/mL) of AAV (AAV-EV or AAV-HMOX1) was injected into NAT10 knockout mice via the tail vein. The fresh specimen section 2 mm below the IVC ligation site was sectioned for hematoxylin and eosin staining.

### Transmission Electron Microscopy

The morphological characteristics of ferroptosis in vascular tissues were analyzed by transmission electron microscopy. Transmission electron microscopy images were captured by a Hitachi HT-7800 transmission electron microscope (Hitachinaka, Ibaraki, Japan).

### Murine Doppler Ultrasound

Mice were anesthetized using a mixture of isoflurane-oxygen for Vascular Doppler ultrasonography. Thrombus formation images were obtained using the Small Animal Ultrasound Imaging System (VINNO6 LAB, VINNO, Suzhou, China).

### Hematoxylin and Eosin Staining

After embedding in paraffin, the tissues were cut into 4 µm-thick sections and stained according to standard procedures. All histological images were observed via light microscopy (E100, Nikon, Japan).

### Enzyme-Linked Immunosorbent Assay

The protein expressions of eNOS (endothelial NO synthase), ET-1 (endothelin-1), TNF-α (tumor necrosis factor-α), and TGF-β1 (transforming growth factor beta 1) were detected following the manufacturer’s instructions with ELISA Kits (Cusabio, Wuhan, China; Multi Sciences, Hangzhou, China).

### Statistical Analysis

The results are expressed as the mean±SEM. Statistical analysis was performed with GraphPad Prism 8.0 software. Before commencing statistical analysis, the data were subjected to tests for normality and equal variance. For comparisons between 2 groups, the 2-tailed Student *t* test was used when the assumptions of normality and homogeneity of variance were satisfied, whereas Welch *t* test was utilized for 2-group analysis with unequal variances. For multiple-group comparisons, we used 1-way ANOVA with Tukey post hoc tests when the assumptions of normality and homogeneity of variance were satisfied, whereas Brown-Forsythe ANOVA with Games-Howell post hoc tests was utilized for multiple-group analysis with unequal variances. The Mann-Whitney *U* test (2 groups) or the Kruskal-Wallis test with Dunn post hoc test (multiple groups) were used when data violated the assumptions of normality or homogeneity of variance. All experiments were replicated independently at least 3 times and statistical significance was ascribed to values of *P*≤0.05.

## Results

### Inhibition of Ferroptosis Ameliorates the Formation of DVT

**Figure 1.**
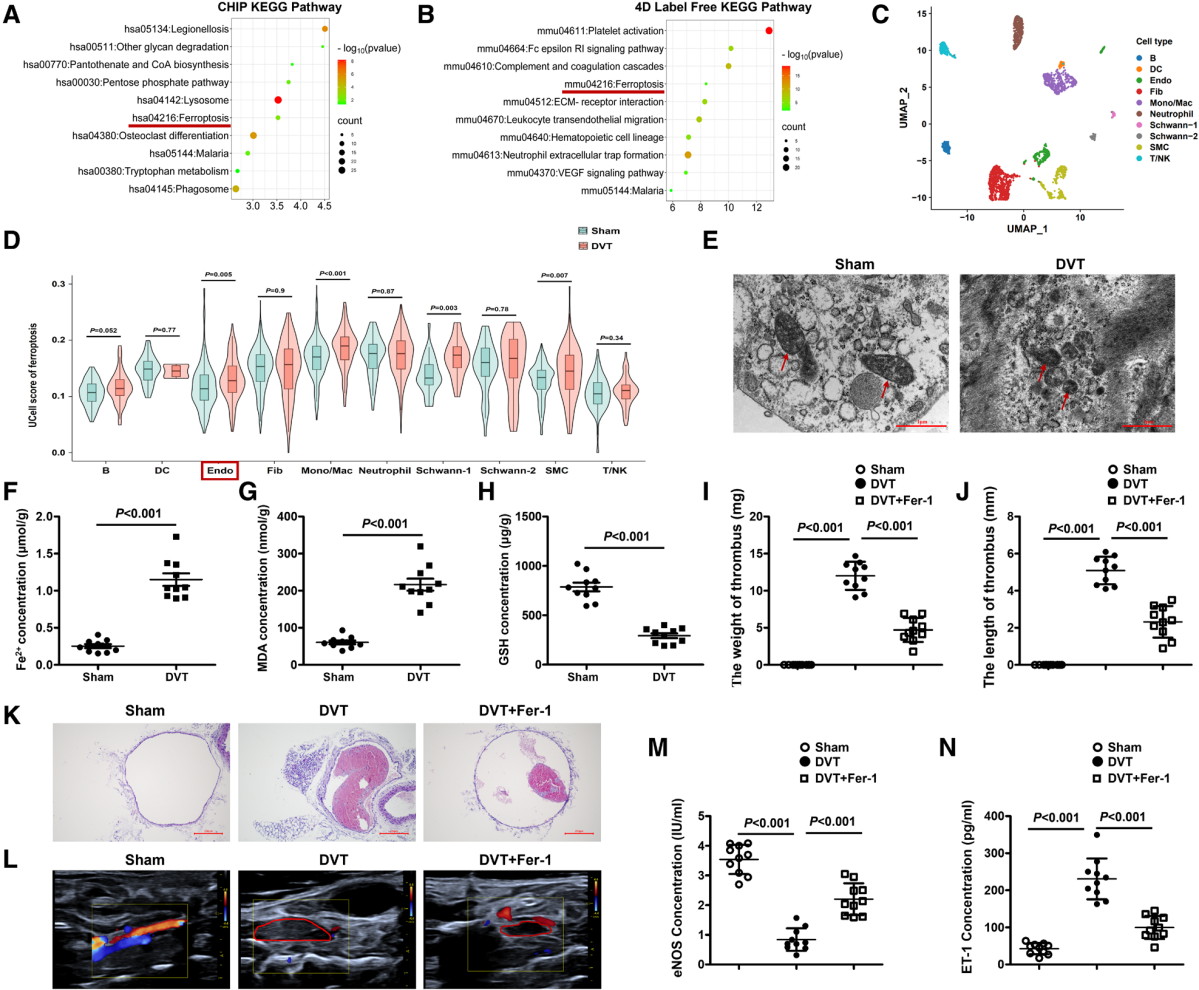
**Inhibition of ferroptosis mitigates the formation of thrombosis in deep vein thrombosis (DVT) mice. A**, The top 10 Kyoto Encyclopedia of Genes and Genomes (KEGG) pathway enrichment analyses according to mRNA microarray analysis of peripheral blood mononuclear cells. **B**, The top 10 KEGG pathway enrichment analyses according to 4-dimensional (4D) Label Free in DVT mice (with thrombus). **C**, Uniform manifold approximation and projection (UMAP) plot of cell clusters presented in the mouse inferior vena cava. **D**, Expression of different cell clusters in ferroptosis. **E**, Representative transmission electron microscopy images showing mitochondrial matrix condensation and the formation of enlarged cristae (red arrowheads) in vascular tissues. Scale bars=1 µm. **F** through **H**, The levels of ferrous ions (Fe^2+^), malondialdehyde (MDA), and glutathione (GSH) in DVT mice (n=10 mice per group). **I** and** J**, Thrombus weights and length are measured in the different treatment groups (n=10 mice per group). **K** and **L**, Representative images of thrombi in each group detected by hematoxylin and eosin (H&E) staining (magnification, ×100) and vascular ultrasound by treating with Fer-1 (ferrostatin-1). Scale bars=200 µm. **M** and **N**, The plasma levels of eNOS (endothelial NO synthase) and ET-1 (endothelin-1) are measured by ELISA after treatment with Fer-1 (n=10 mice per group). Results are expressed as mean±SEM. Statistical analysis was performed by 2-tailed Student *t* test (**D** and **H**), Welch *t* test (**F** and **G**), 1-way ANOVA with Tukey post hoc tests (**M**), and Brown-Forsythe ANOVA with Games-Howell post hoc tests (**I, J,** and** N**). CoA indicates coenzyme A; DC, dendritic cell; ECM, extracellular matrix; Endo, endothelial cell; Mono/Mac, monocyte/macrophage; RI, receptor I; SMC, smooth muscle cell; T/NK, T lymphocyte/natural killer cell; and VEGF, vascular endothelial growth factor.

To elucidate the pathological factors involved in DVT formation, we collected the peripheral blood mononuclear cells from DVT patients in the clinic and the thrombus of the vein from the DVT model mice, and performed CHIP (GSE307102, *P*<0.05, FC>1.5) and 4D Label Free proteomic analysis (PXD067933, *P*<0.05, FC>2). By Kyoto Encyclopedia of Genes and Genomes analysis according to the differentially expressed genes and proteins revealed by CHIP and 4D Label Free data, the ferroptosis pathway was found to be significantly enriched for both analyses (Figure 1A and 1B). In addition, single-cell RNA sequencing data (GSE221978) demonstrated a significant enrichment of endothelial ferroptosis in DVT (Figure 1C and 1D). Interestingly, morphological examination revealed shrunken mitochondria and reduced cristae in the vascular endothelium of DVT mice (Figure 1E). The levels of Fe^2+^ and malondialdehyde were significantly elevated in DVT mice (Figure 1F through 1G), whereas glutathione levels were decreased (Figure 1H). Similarly, the FeCl_3_-induced injury model showed elevated malondialdehyde and decreased glutathione levels (Figure S11I through S11J). To further validate the predominant role of ferroptosis in DVT formation, we administered Fer-1 intraperitoneally before DVT model induction. As anticipated, treatment with Fer-1 resulted in a substantial reduction in thrombus size and alleviated the vascular endothelial dysfunction induced by ferroptosis in DVT (Table S6; Figure 1I through 1N). Furthermore, we generated endothelial cell–specific GPX4-knockout mice by crossing GPX4^fl/+^ mice with Cdh5-Cre^ERT2^ mice. In the vascular endothelium of GPX4^fl/fl^Cdh5-Cre^+^ mice, both mRNA and protein levels of GPX4 were markedly decreased (Figure S1A and S1B). Notably, GPX4 silencing exacerbated the formation of thrombosis and the vascular endothelial dysfunction in DVT (Figure S1C through S1G). These findings suggest that ferroptosis plays a pivotal role in the pathogenesis of DVT.

### NAT10-Mediated Ferroptosis Exacerbates the Formation of Thrombosis

**Figure 2.**
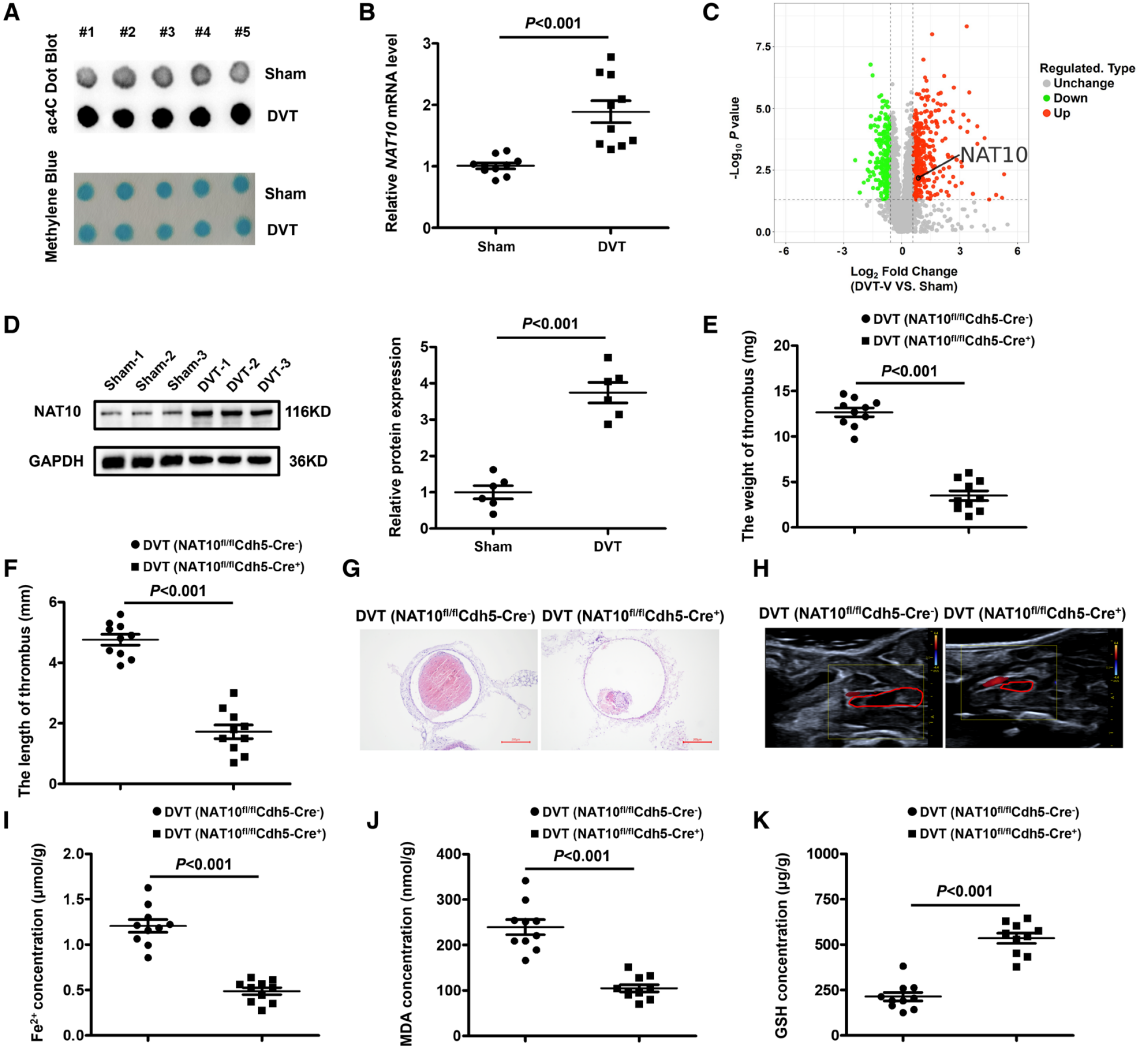
**Silencing of NAT10 (*N*-acetyltransferase 10) significantly attenuates ferroptosis in deep vein thrombosis (DVT) mice. A**, Dot blotting shows that the total *N*4-acetylcytidine (ac4C) levels of vascular endothelium are higher in DVT mice (n=5 mice per group). Methylene blue staining was used as loading control. **B**, Relative expression levels of NAT10 mRNA are determined by quantitative real-time polymerase chain reaction in DVT mice (n=10 mice per group). **C**, Volcano plot showing the profiling data of upregulated NAT10 in the vascular tissues of 6 DVT mice (without thrombus) compared with that of 6 controls determined by 4-dimensional label free. **D**, The protein levels of NAT10 are detected by Western blotting (n=6 mice per group). **E** and **F**, Thrombus weights and length are measured in the different treatment groups (n=10 mice per group). **G** and **H**, Representative images of thrombi in DVT mice with NAT10 knockdown were detected by hematoxylin and eosin staining (magnification, ×100) and vascular ultrasound. Scale bars=200 µm. **I** through** K**, The levels of ferrous ions (Fe^2+^), malondialdehyde (MDA), and glutathione (GSH) in NAT10 knockdown of DVT mice (n=10 mice per group). Results are expressed as mean±SEM. Statistical analysis was performed by 2-tailed Student *t* test (**D** through **F, I,** and** K**), Welch *t* test (**B** and **J**).

To test whether ac4C modification participates in the process of DVT formation, we first performed dot blotting and found that the total ac4C modification of RNA was significantly elevated in the vascular endothelium of DVT mice (Figure 2A). Consistently, NAT10, functioning as an ac4C writer, exhibited markedly higher expression in the vascular endothelium of DVT mice and the FeCl_3_-induced injury model (Figure 2B; Figure S11G and S11H). In addition, 4D Label Free data analysis indicated that NAT10 was upregulated in DVT mice (Figure 2C), which was also confirmed in the vascular endothelium of DVT mice (Figure 2D). These results implied that NAT10 might function in the pathogenesis of DVT.

To validate the function of NAT10 in DVT, we pretreated DVT mice with Remodelin (a small molecule inhibitor of NAT10) and found that the NAT10 inhibitor ameliorated the formation of thrombosis (Figure S2). Meanwhile, the NAT10 inhibitor markedly attenuated both endothelial damage and ferroptosis in the FeCl_3_-induced injury model (Figure S11K through S11M). To assess the role of NAT10 in endothelial ferroptosis, we established endothelial cell–specific NAT10-knockout mice by crossing NAT10^fl/+^ mice with Cdh5-Cre^ERT2^ mice. NAT10 mRNA and protein levels in NAT10^fl/fl^Cdh5-Cre^+^ mice were notably decreased in vascular endothelium compared with their NAT10^fl/fl^Cdh5-Cre^-^ littermate controls (Figure S3). As expected, NAT10 knockout significantly alleviated the formation of thrombosis (Figure 2E through 2H). Moreover, inhibition of NAT10 was shown to alleviate ferroptosis in the endothelial cells of DVT mice (Figure 2I through 2K). Collectively, these findings demonstrate that inhibition of NAT10 reduces ferroptosis in endothelial cells and helps alleviate the symptoms of DVT.

### Downregulation of NAT10 Enhances Ferroptosis Resistance In Vitro

To further illustrate the role of NAT10 in regulating endothelial injury and ferroptosis, we induced endothelial dysfunction with Ang II in HUVECs, which significantly upregulated NAT10 and HMOX1 expression and increased malondialdehyde levels (Figure S11A through S11C). Meanwhile, it also inhibited the expression of glutathione (Figure S11D). Subsequently, we used 2 distinct ferroptosis inducers (RSL3, FINO2) to trigger ferroptosis and found that both inducers significantly increased the expression of NAT10 protein (Figure S4D). However, Fer-1 attenuated the Ang II–induced upregulation of NAT10 (Figure S11E and S11F).

**Figure 3.**
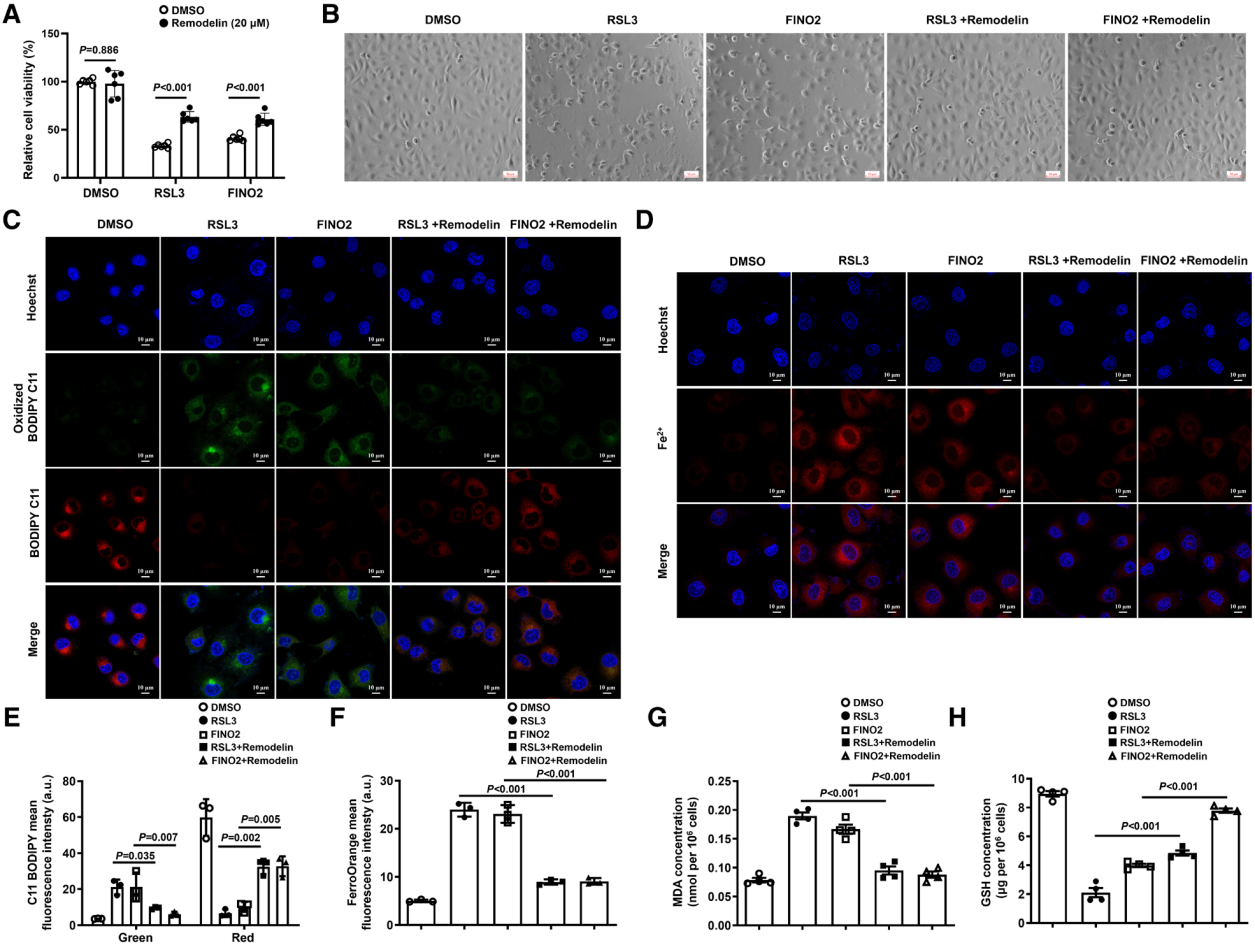
**Inhibition of NAT10 (*N*-acetyltransferase 10) protects against ferroptosis in human umbilical vein endothelial cells (HUVECs). A**, Cell viability is detected with CCK8 assay kit (n=6). **B**, Images of brightfield, the HUVECs are treated with RSL3 (4 μM)/FINO2 (40 μM) or remodelin (20 μM) for 12 hours (magnification, ×100). **C** and **E**, Lipid peroxidation detected by BODIPYTM 581/591 C11 probe inhibits NAT10, followed by RSL3 (4 μM) or FINO2 (40 μM) treatment for 12 hours in HUVECs (magnification, ×600). Green images represent oxidized lipids, while red images represent nonoxidized lipids. Scale bars=10 µm. **D** and** F**, FerroOrange probes showed ferrous ions (Fe^2+^) after inhibiting NAT10, followed by RSL3 or FINO2 treatment for 12 hours in HUVECs (magnification, ×600). Red in images represents Fe^2+^; blue represents the nucleus. Scale bars=10 µm. **G** and **H**, The levels of malondialdehyde (MDA) and glutathione (GSH) in HUVECs (n=4). Results are expressed as mean±SEM. The n refers to the number of biological replicates. All experiments were independently repeated at least 3×. Statistical analysis was performed by 2-tailed Student *t* test (RSL3 and FINO2 of **A**), Welch *t* test (dimethyl sulfoxide [DMSO] of **A**), and 1-way ANOVA with Tukey post hoc tests (**E** through **H**).

In addition, we assessed the suppressive effects of NAT10 inhibition in HUVECs and C166 cells, thereby elucidating the role of NAT10 in ferroptosis. Inhibition of NAT10 remarkably alleviated the decrease in cell viability induced by RSL3 and FINO2 (Figure 3A; Figure S4A through S4C and S5A and S5B). Furthermore, the morphological alterations distinct for ferroptosis were significantly reversed on inhibition of NAT10 (Figure 3B). From the perspective of molecular levels for the regulation of ferroptosis, we found downregulation of NAT10 led to a significant reduction in the levels of lipid peroxidation and Fe^2+^ that were induced by RSL3 and FINO2 (Figure 3C through 3F; Figure S5D and S5E). In addition, we also observed that inhibition of NAT10 resulted in decreased malondialdehyde levels (Figure 3G) and an increase in glutathione abundances (Figure 3H; Figure S5C). In summary, these data indicate that NAT10 deficiency significantly impedes ferroptosis in vitro.

### NAT10 Stabilizes HMOX1 by Inducing Its ac4C Modification

**Figure 4.**
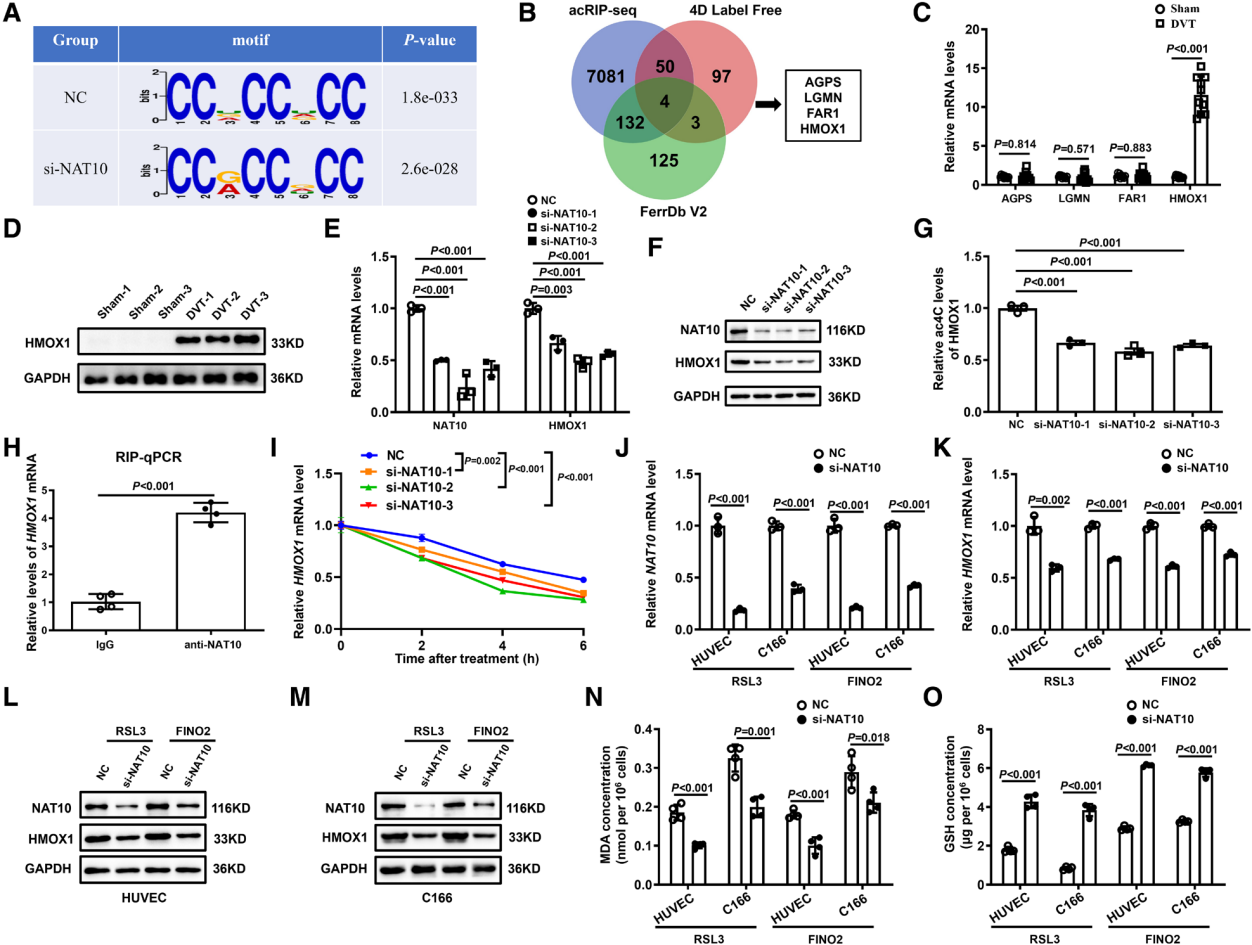
**NAT10 (*N*-acetyltransferase 10) mediates HMOX1 (heme oxygenase 1) mRNA acetylation in vitro. A**, Acetylated RNA immunoprecipitation sequencing (acRIP-seq) analysis of the highly enriched motif within *N*4-acetylcytidine (ac4C) peaks. **B**, The Venn diagram shows that mRNAs are predicted by acRIP-seq, 4-dimensional (4D) Label Free, and FerrDb V2 Database. **C**, Levels of mRNAs in deep vein thrombosis (DVT) mice compared with control subjects are measured by quantitative real-time polymerase chain reaction (qRT-PCR; n=10). **D**, The expression of HMOX1 is determined by Western blotting in DVT mice. **E** and **F**, The NAT10 and HMOX1 mRNA and protein levels are detected by qRT-PCR and Western blotting in si-NAT10s. **G**, The ac4C modification level of HMOX1 mRNA is detected by acRIP-qPCR (acetylated RNA immunoprecipitation-quantitative PCR; n=3). **H**, RIP-qPCR assay verifies the interaction between NAT10 and HMOX1 (n=4). **I**, Stability of HMOX1 is detected after treatment with actinomycin D for the indicated times (n=3). **J** through **M**, The mRNA and protein levels of NAT10 and HMOX1 are determined by qRT-PCR and Western blotting in human umbilical vein endothelial cells (HUVECs) and C166 cells of each treatment group. **N** and **O**, The levels of malondialdehyde (MDA) and glutathione (GSH) are measured in HUVECs and C166 cells (n=4). Results are expressed as mean±SEM. The n refers to the number of biological replicates. All experiments were independently repeated at least 3×. Statistical analysis was performed by 2-tailed Student *t* test (**H, J, K, N,** and** O**), Welch *t* test (**C**), 1-way ANOVA with Tukey post hoc tests (**E** and **G**), and 2-way ANOVA (**I**). acRIP-qPCR indicates acetylated RNA immunoprecipitation-quantitative PCR; AGPS, alkyldihydroxyacetonephosphate synthase; FAR1, fatty acyl-CoA reductase 1; LGMN, legumain; NC, negative control; RIP-qPCR, RNA immunoprecipitation-quantitative PCR; and siNAT10, small interfering RNA N-acetyltransferase 10.

To investigate the mechanisms underlying the role of NAT10 in modulating the functions of vascular endothelial cells, we performed acRIP-seq to reveal the differentially modified mRNAs on NAT10 knockdown in HUVECs. Sequential analysis of ac4C modifications showed a significant enrichment of typical CXXCXXCXX motifs within the ac4C sites (Figure 4A). Consistent with prior studies, we observed that ac4C peaks were predominantly localized within the coding sequences of mRNA transcripts in both negative control and si-NAT10 groups (Figure S6). Given that NAT10 enhances ac4C modification and RNA stability, we utilized Venn diagrams to analyze NAT10 targets from 3 sources: 4D Label Free (FC>2; *P*<0.05), FerrDb V2 Database (Driver), and downregulated genes of acRIP-seq (FC>2; *P*<0.05). As a result, AGPS (alkyldihydroxyacetonephosphate synthase), LGMN (legumain), FAR1 (fatty acyl-CoA reductase 1), and HMOX1 were screened out from the overlap (Figure 4B), and among these genes, HMOX1 exhibited a notable upregulation in mice with DVT (Figure 4C and 4D). Subsequent assessments revealed that the mRNA and protein levels of HMOX1 were markedly diminished after NAT10 knockdown, whereas HMOX1 was significantly increased after overexpression of NAT10 (Figure 4E and 4F; Figures S7, S10A, and S10B). In addition, acRIP-qPCR (acetylated RNA immunoprecipitation-quantitative PCR) results demonstrated a consistent decrease in the levels of ac4C-modified HMOX1 mRNA in si-NAT10 HUVECs (Figure 4G). Furthermore, RIP-qPCR analysis confirmed that NAT10 could bind to HMOX1 mRNA (Figure 4H). Notably, the degradation of HMOX1 mRNA induced by actinomycin D was exacerbated by the inhibition of NAT10 (Figure 4I). Based on these findings, we speculate that NAT10 regulates ferroptosis by acetylating HMOX1 mRNA.

Then, the functional consequences of NAT10 knockdown-mediated inhibition of HMOX1 were systematically investigated. Our findings provided evidence that the suppression of NAT10 led to a pronounced reduction in the expression of HMOX1 (Figure 4J through 4M). Moreover, inhibition of NAT10 was accompanied by a marked decrease in malondialdehyde levels and a concomitant increase in glutathione under ferroptosis conditions (Figure 4N and 4O).

Further analysis revealed that si-NAT10 treatment could significantly suppress the expression of ICAM1 (intercellular adhesion molecule 1), SELP (selectin P), and TNF-α while increasing the levels of eNOS and TGF-β1 in HUVECs (Figure S8). Overall, these findings suggest that NAT10 enhances the stability of HMOX1 mRNA via ac4C modification, thereby playing a crucial role in the regulation of endothelial cell function.

### Inhibition of NAT10 Restores Endothelial Function via Suppression of HMOX1

**Figure 5.**
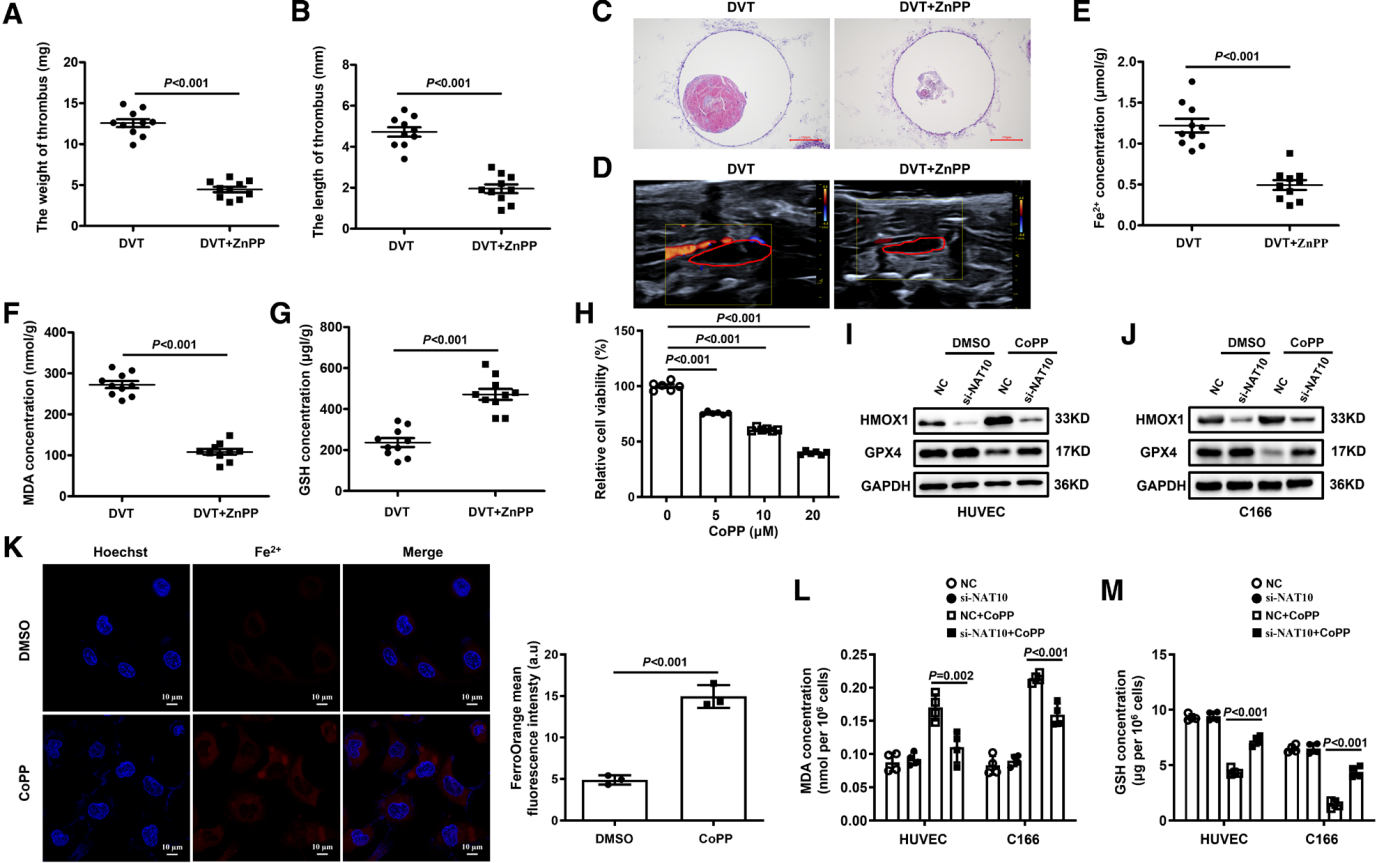
**Inhibition of NAT10 (*N*-acetyltransferase 10) expression alleviates ferroptosis by downregulating HMOX1 (heme oxygenase 1). A** and **B**, Thrombus weights and length are measured in the different treatment groups (n=10 mice per group). **C** and **D**, Representative images of thrombi in deep vein thrombosis (DVT) mice treated with ZnPP (zinc protoporphyrin IX; an HMOX1 inhibitor) were detected using hematoxylin and eosin staining (magnification, ×100) and vascular ultrasound. Scale bars=200 µm. **E** through **G**, The levels of ferrous ions (Fe^2+^), malondialdehyde (MDA), and glutathione (GSH) in DVT mice (n=10 mice per group). **H**, Cell viability is detected with CCK8 assay kit in human umbilical vein endothelial cell (HUVECs; n=6). **I** and **J**, The HMOX1 and GPX4 (glutathione peroxidase 4) protein levels are detected by Western blotting in si-NAT10 (with 20 μM CoPP [cobalt protoporphyrin IX] treatment). **K**, Fe^2+^ levels of HUVECs after inhibiting NAT10 followed by CoPP treatment for 12 hours. Red in images represents Fe^2+^; blue represents the nucleus (magnification, ×600). Scale bars=10 µm. **L** and **M**, The levels of MDA and GSH are measured in si-NAT10 (with 20 μM CoPP treatment; n=4). Results are expressed as mean±SEM. The n refers to the number of biological replicates. All experiments were independently repeated at least 3 times. Statistical analysis was performed by 2-tailed Student *t* test (**A, B, E** through **G**, and** K**) and 1-way ANOVA with Tukey post hoc tests (**H, L,** and** M**). CCK8 indicates cell counting kit 8; DMSO, dimethyl sulfoxide; NC, negative control; and siNAT10, small interfering RNA N-acetyltransferase 10.

Previous studies have indicated that the upregulation of HMOX1 is associated with increased ferroptosis in diabetic human endothelial cells.^26^ To clarify the functional mechanism of HMOX1 in DVT formation, we first checked the 4D Label Free proteomics data and found that HMOX1 was significantly overexpressed in DVT mice (Figure S9A). To determine whether HMOX1 influences DVT formation, we injected ZnPP into mice to inhibit HMOX1 function.^36^ Histological analysis via hematoxylin and eosin staining and Doppler imaging showed a concurrent reduction in thrombus size of DVT mice treated with ZnPP (Figure 5A through 5D). Meanwhile, the levels of Fe^2+^ and malondialdehyde were decreased, whereas glutathione was increased in DVT mice treated with ZnPP (Figure 5E through 5G).

In addition, to further elucidate the specific role of HMOX1, we utilized a selective inducer of HMOX1 known as CoPP, which effectively elevated HMOX1 levels.^37^ The results demonstrated that specific overexpression of HMOX1 could significantly inhibit the cell viability in HUVECs (Figure 5H). Meanwhile, overexpression of HMOX1 dramatically increased the levels of Fe^2+^, ICAM1, SELP, and TNF-α and inhibited the expressions of eNOS and TGF-β1 in vitro (Figure 5K; Figure S9B). Notably, the overexpression of HMOX1 led to iron accumulation, which further aggravated ferroptosis in endothelial cells.

Although we have identified that HMOX1 is a downstream target of NAT10, further evidence is needed to demonstrate that NAT10 affects ferroptosis by regulating HMOX1. Therefore, we overexpressed HMOX1 in si-NAT10 cells and interfered with HMOX1 in Ad-NAT10 cells. Intriguingly, HMOX1 overexpression increased cell ferroptosis, whereas inhibition of NAT10 could reduce malondialdehyde content and increase the expression of glutathione and GPX4, which led to a reduction in ferroptosis (Figure 5I, 5J, 5L, and 5M). Consistently, silencing HMOX1 with siRNA significantly promoted the expression of glutathione and GPX4 in Ad-NAT10 cells (Figure S10C through S10F). Together, these data indicated that inhibition of NAT10 reduced HMOX1 expression and alleviated ferroptosis, thereby attenuating endothelial dysfunction.

### NAT10 Alleviates the Formation of DVT by Regulating the HMOX1

**Figure 6.**
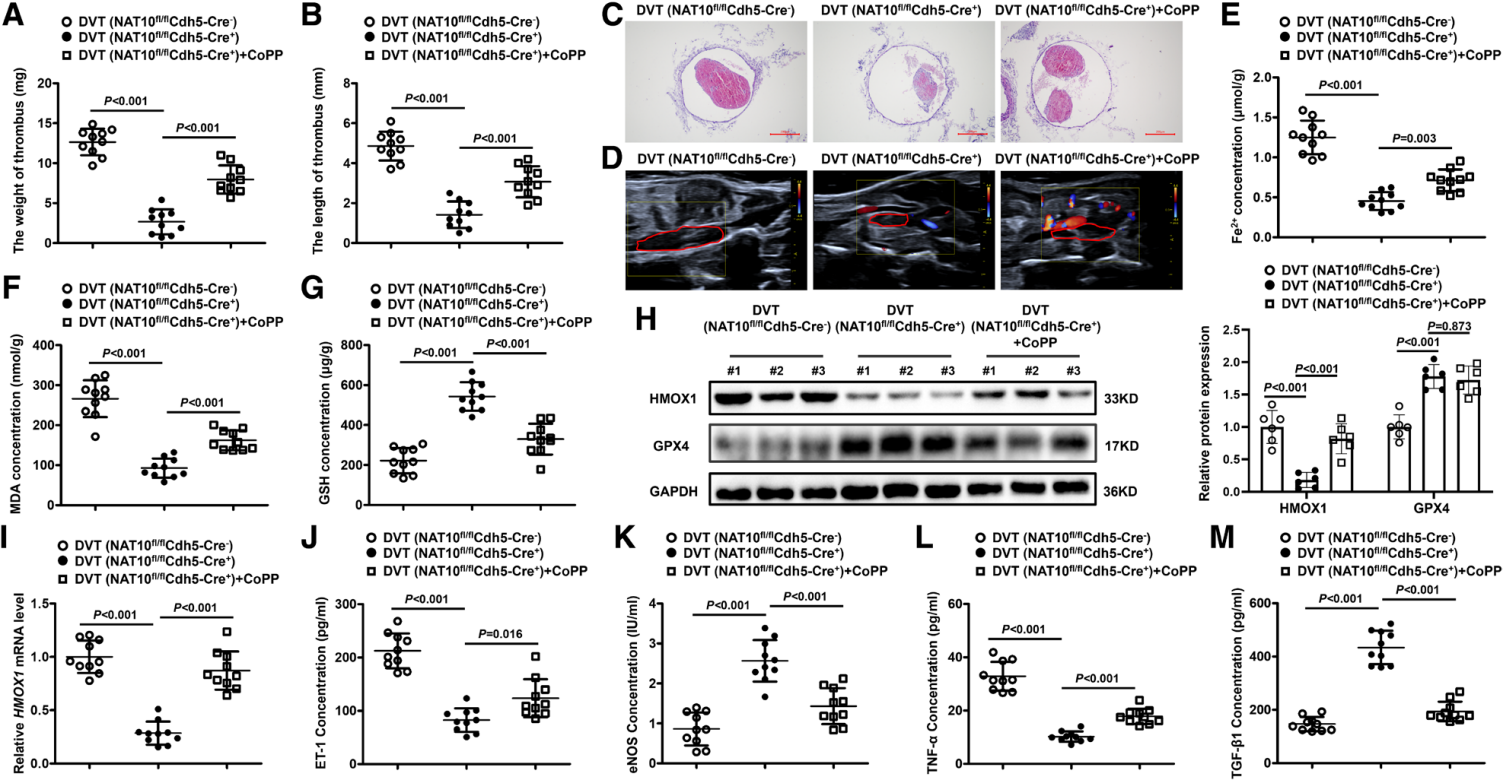
**Knockout NAT10 (*N*-acetyltransferase 10) represses HMOX1 (heme oxygenase 1) to reduce the formation of deep vein thrombosis (DVT) in vivo. A** and **B**, Thrombus weights and length are measured in the different treatment groups (n=10 mice per group). **C** and **D**, Representative images of thrombi in NAT10^fl^^/fl^Cdh5-Cre^+^ (NAT10 knockdown) mice were detected by hematoxylin and eosin staining (magnification, ×100) and vascular ultrasound. Scale bars=200 µm. **E** through **G**, Relative expression levels of ferrous ions (Fe^2+^), malondialdehyde (MDA), and glutathione (GSH) in different treatment groups (n=10 mice per group). **H**, Expressions of HMOX1 and GPX4 (glutathione peroxidase 4) are determined by Western blotting in different treatment groups (n=6 mice per group). **I**, The expression of HMOX1 is determined by quantitative real-time polymerase chain reaction (qRT-PCR) in different treatment groups (n=10 mice per group). **J** through **M**, Expression of ET-1 (endothelin-1), eNOS (endothelial NO synthase), TNF-α (tumor necrosis factor-α), or TGF-β1 (transforming growth factor beta 1) are determined by ELISA in the plasma of each treatment group (n=10 mice per group). Results are expressed as mean±SEM. Statistical analysis was performed by 1-way ANOVA with Tukey post hoc tests (**A, B, E** through **L**) and Brown-Forsythe ANOVA with Games-Howell post hoc tests (**M**). CoPP indicates cobalt protoporphyrin IX.

To validate the role of NAT10 in modulating HMOX1 to alter DVT formation, we injected CoPP into NAT10-knockout mice to activate HMOX1 function. Similarly, we constructed an AAV vector to overexpress HMOX1 under the control of the specific TIE promoter in vascular endothelium (Figure S12D). Based on the results of hematoxylin and eosin staining and Doppler, we found that thrombus size was decreased in DVT NAT10-knockout mice, whereas HMOX1 overexpression reversed the effects of NAT10 deficiency (Figure 6A through 6D; Figure S12B and S12C). In addition, NAT10 silencing significantly reduced the levels of Fe^2+^ and malondialdehyde, whereas HMOX1 overexpression induced an increase in such ferroptosis markers (Figure 6E and 6F; Figure S12E). Meanwhile, NAT10 deficiency also increased the levels of glutathione and GPX4 (Figure 6G and H; Figure S12F). Notably, NAT10 silencing was associated with decreased expression of HMOX1 (Figure 6H and 6I; Figure S12D). Moreover, Knockdown of NAT10 significantly decreased the levels of ET-1 and TNF-α, and increased the levels of eNOS and TGF-β1 in DVT mice (Figure 6J through 6M; Figure S12A). Similarly, the mRNA levels of ICAM1 and SELP were remarkably decreased in NAT10-knockout mice (Figure S12A). All these data suggest that inhibition of NAT10 reduces HMOX1 expression and attenuates ferroptosis, thereby mitigating endothelial dysfunction and the formation of thrombosis in DVT mice.

## Discussion

Ferroptosis is a form of iron-dependent programmed cell death that has been implicated in various vascular pathologies, including cardiovascular, cerebrovascular, and peripheral vascular diseases.^38,39^ Notably, DVT, as a prevalent peripheral vascular condition, is characterized by a multifaceted interplay of factors, such as blood flow, endothelial injury, and inflammatory response.^40,41^ In accordance with previous studies, it is evident that ferroptosis is crucial in the formation of thrombogenesis.^8^ Through the generation of vascular endothelial cell conditional GPX4 knockout mice, we confirmed that the knockout of GPX4 substantially aggravated thrombosis. Hence, identifying key targets to inhibit endothelial ferroptosis is of great significance for the repair of endothelial injury. However, the mechanisms governing endothelial ferroptosis, especially the upstream regulatory molecules, remain poorly understood. Given the pivotal role of endothelial ferroptosis in DVT, we aimed to identify the upstream regulatory network of endothelial ferroptosis in DVT.

Iron-mediated Fenton reactions generate reactive oxygen species that oxidize polyunsaturated fatty acids in cell membranes. The subsequent oxidative cleavage of polyunsaturated fatty acids yields malondialdehyde, making its elevated levels a reliable indicator of ferroptosis progression and oxidative damage.^42^ Apart from lipid peroxidation inhibitors and iron chelators, mounting evidence showed that ac4C modification of mRNA may also regulate ferroptosis. The research field of ac4C modification is currently garnering increasing attention as it has been intricately associated with many critical biological processes, including osteogenic differentiation, cancer cell progression, and cardiac remodeling.^43^ Despite the fact that DVT is characterized by dysregulated epigenetic mechanisms, the involvement of ac4C modification in DVT has yet to be reported.

Notably, NAT10 is the first enzyme identified to catalyze ac4C modification and possesses both acetyltransferase and RNA-binding activity. In particular, NAT10 functions as a writer for ac4C, controlling the expression of its target genes involving RNA stability.^44^ NAT10 promotes malignant progression in multiple myeloma by acetylating centrosomal protein 170 mRNA to enhance its translation and drive cell proliferation.^45^ Previous research has indicated that NAT10 mediated ac4C modification of MDM2 transcript, which subsequently led to the stabilization of its mRNA, and finally caused progression of the disease in gastric cancer.^46^ In addition, NAT10 has also been found to enhance the ac4C modification of NCOA4 (nuclear receptor coactivator 4) mRNA, which contributes to ferroptosis induced by ischemia-reperfusion injury in tubular epithelial cells.^47^ Notably, although our study demonstrates that NAT10 promotes ferroptosis in endothelial cells, previous studies have reported that NAT10 can exacerbate cardiac ischemia-reperfusion injury by promoting ferroptosis in cardiomyocytes and promote colon cancer cell proliferation by stabilizing ferroptosis suppressor protein 1 mRNA to suppress ferroptosis.^21,48^ This contrast highlights that the regulatory role of NAT10 in ferroptosis may exhibit tissue and disease-specific differences. Despite the growing body of evidence highlighting the influence of NAT10-dependent ac4C modification across various diseases, the role of NAT10 in DVT remains poorly understood.

Our current study demonstrated that NAT10 expression was significantly upregulated in the venous vascular tissues of DVT mice, with a positive correlation between NAT10 levels and disease severity. Strikingly, inhibition of NAT10 attenuates the formation of thrombosis in DVT mice. Because these inhibitors may participate in multiple cellular pathways, their suppression will disrupt nontarget biological processes. Moreover, their inhibitory effects are likely systemic rather than tissue-specific, potentially causing side effects due to widespread exposure. To further elucidate the role of NAT10, we generated endothelial-specific NAT10 knockdown mice. Using this murine model, we found that NAT10 silencing emerged as a potential therapeutic strategy for the prevention and treatment of DVT. Besides, NAT10 silencing was shown to mitigate DVT formation by decreasing ferroptosis in endothelial cells. The combined analysis of acRIP-seq provided the evidence that NAT10-mediated ac4C modification regulates the stability of HMOX1 mRNA, contributing to the aberrant upregulation of HMOX1 in DVT. Hence, it is hypothesized that NAT10 is capable of facilitating ferroptosis by upregulating HMOX1, leading to iron overload and inhibiting the generation of lipid peroxides. These findings offer a novel perspective on the functional role of NAT10-mediated ac4C modification in the pathogenesis of DVT.

Combining the current data and previous research, HMOX1 plays a critical role in the regulation of ferroptosis in endothelial cells by increasing iron levels, which subsequently leads to enhanced production of ROS and lipid peroxidation.^49,50^ Moreover, our present study also indicates that HMOX1 facilitates the release of free iron, thereby promoting ferroptosis and contributing to thrombus formation. More importantly, we observed a significant upregulation of HMOX1 in DVT, and the inhibition of HMOX1 resulted in decreased incidence of thrombosis. Furthermore, the knockdown of NAT10, which further suppressed HMOX1 expression, not only diminished iron levels but also reduced lipid peroxidation, thereby ameliorating ferroptosis in endothelial cells.

In conclusion, this study identified NAT10 as a pivotal regulator of ferroptosis in DVT. We provided strong evidence that NAT10 enhances the stability of HMOX1 through ac4C modification, which results in iron overload and lipid peroxides, thereby forming a positive feedback loop that exacerbates DVT. Our findings indicate that targeting NAT10 could be a promising therapeutic strategy for inhibiting ferroptosis and restoring the function of endothelial cells.

## ARTICLE INFORMATION

### Acknowledgments

The authors thank Qiang Zou (Shanghai Jiao Tong University School of Medicine) for providing constructive suggestions.

### Sources of Funding

This study was assisted by the National Natural Science Foundation of China (81673981, 82274575, and 82575078), Major Basic Research Project of Shandong Natural Science Foundation (ZR2023ZD56), Science & Technology Cooperation Program of Shandong (2025KJHZ035), the Natural Science Foundation of Shandong Province (ZR2022LZY011), Coconstruction project of State Administration of TCM (GZY-KJS-SD-2023-034, GZY-KJS-SD-2023-046), Taishan Scholars Program (tstp20240513), the Shandong Province Taishan Scholar Project (No.tsqn202306393), and Central Government Guides Local Science and Technology Development Fund Projects of Shandong Province (YDZX20203700001407), Undergraduate Education Reform Project of Shandong Province (Z2024231), Key Laboratory of TCM Classical Theory, Ministry of Education, Open Research Project, National Youth Qihuang Scholar Training Program and Shandong Province TCM High Level Talent Cultivation Project.

### Disclosures

None.

### Supplemental Material

Supplemental Methods

Tables S1–S6

Figures S1–S12

Major Resources Table

ARRIVE Guideline

Full Unedited Western Blot

## Supplementary Material
