# Supplementary material for: NAT10-Mediated ac4C-Modification Exacerbates Ferroptosis by Stabilizing HMOX1 in Deep Vein Thrombosis
Source: Arterioscler Thromb Vasc Biol. 2025 Dec 30;46(2):e323986. doi: 10.1161/ATVBAHA.125.323986 (PMC12822780; doi:10.1161/ATVBAHA.125.323986)
Supplement: Supplementary file 1 [file atv-46-e323986-s001.pdf]

Figure 2D

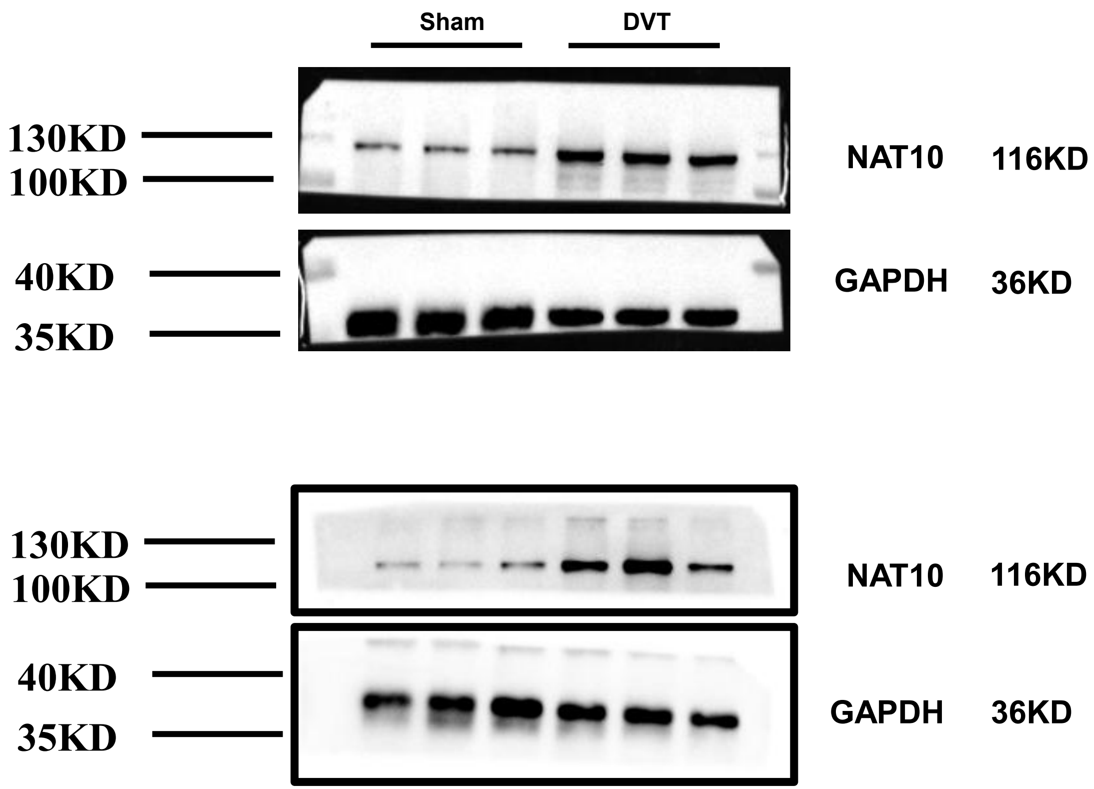

The same membrane were cut to blot for different proteins.

Figure 4D

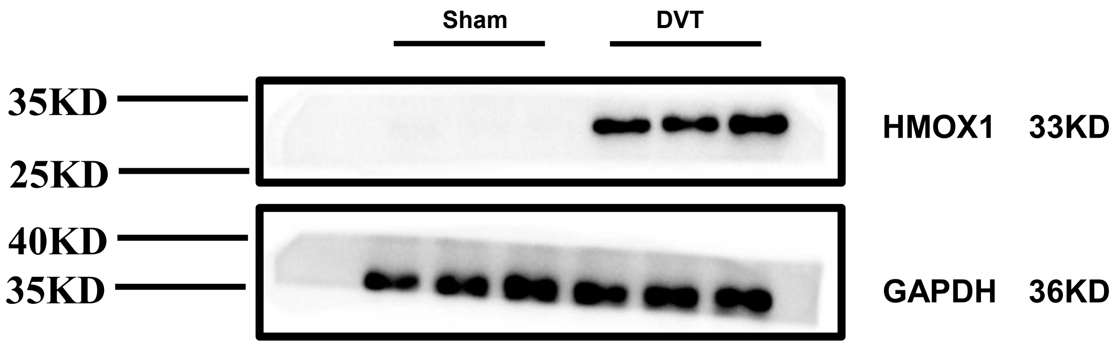

The same membrane were cut to blot for different proteins.

Figure 4F

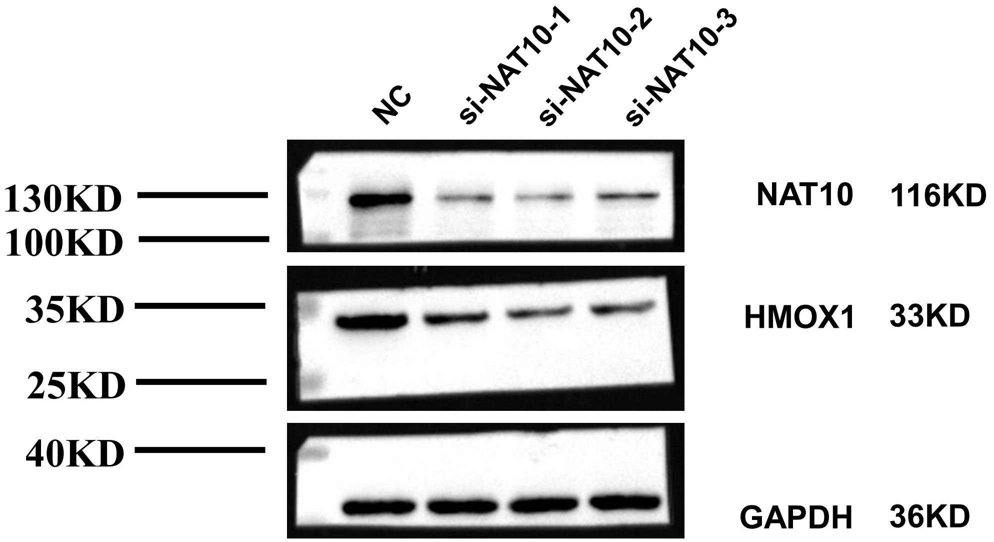

The same membrane were cut to blot for different proteins.

Figure 4L

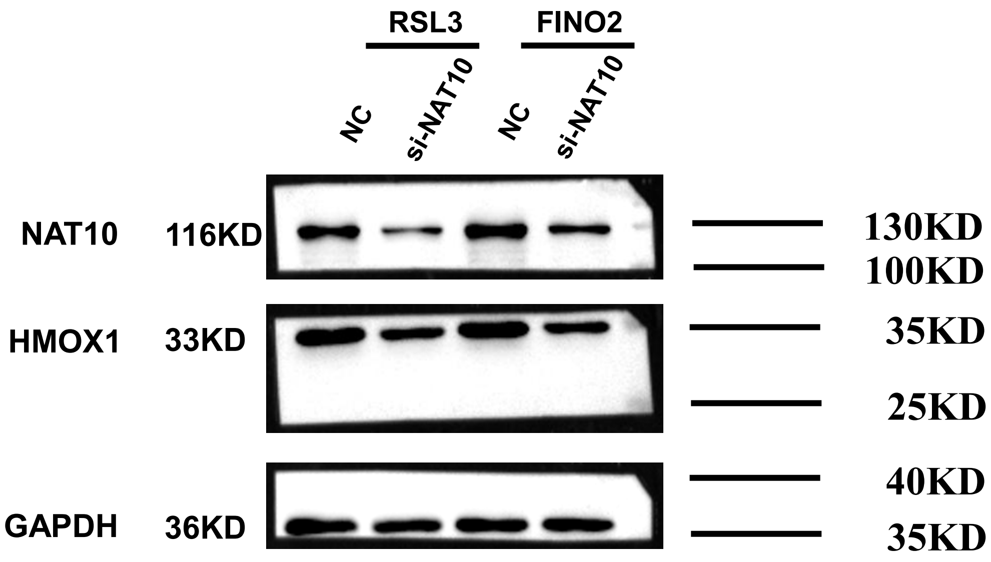

The same membrane were cut to blot for different proteins.

Figure 4M

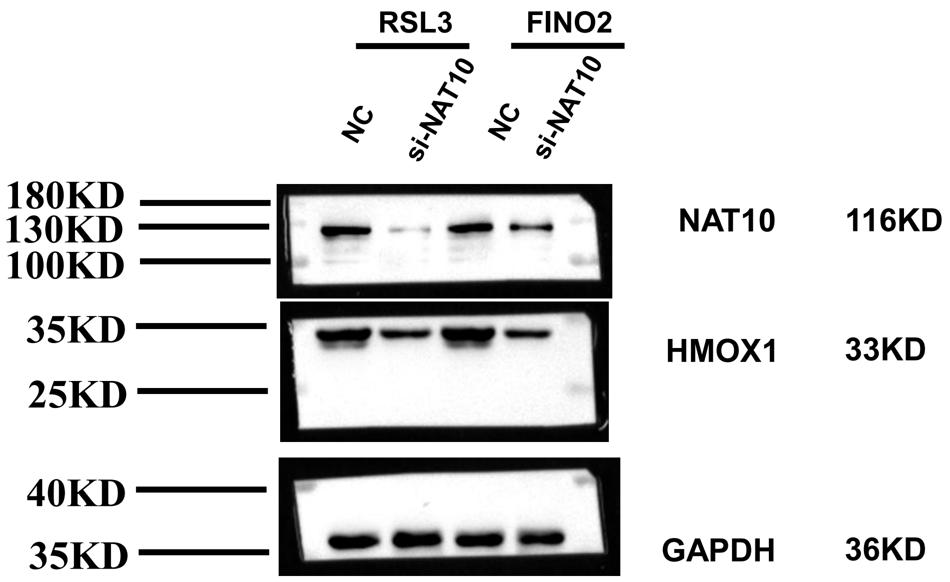

The same membrane were cut to blot for different proteins.

Figure 5I

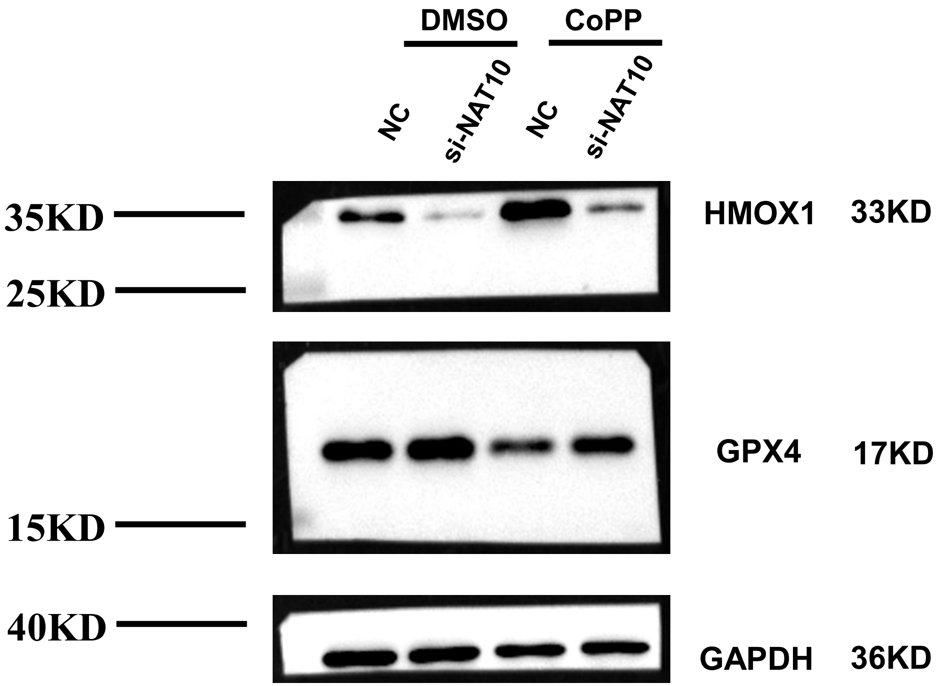

The same membrane were cut to blot for different proteins.

Figure 5J

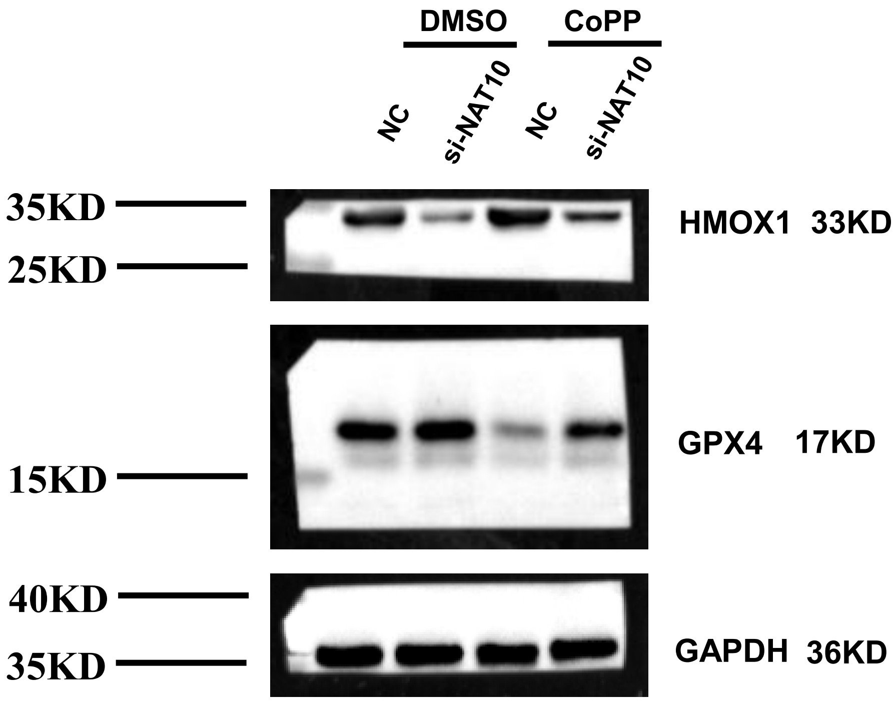

The same membrane were cut to blot for different proteins.

Figure 6H

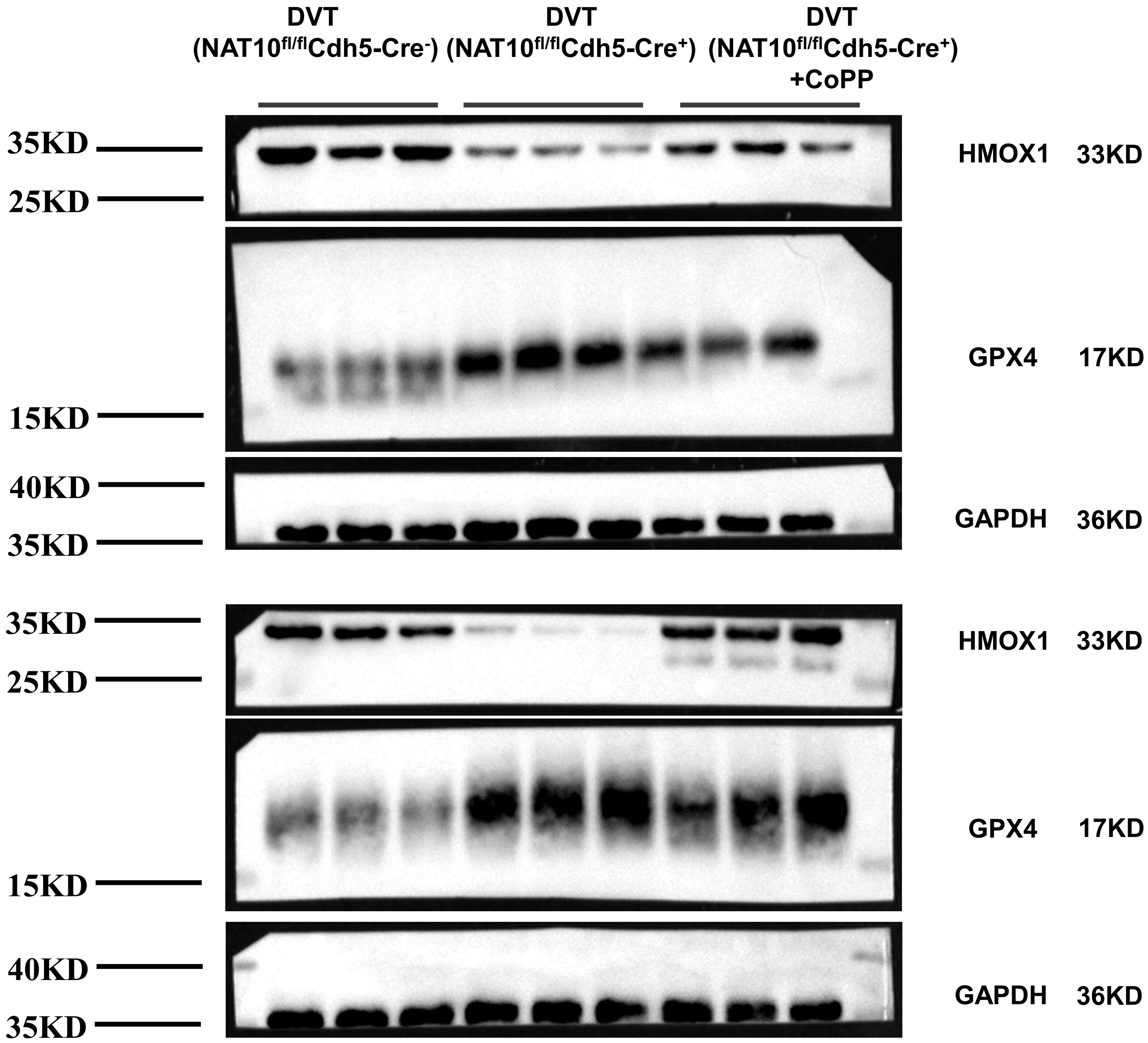

The same membrane were cut to blot for different proteins.

Supplementary Figure 1B

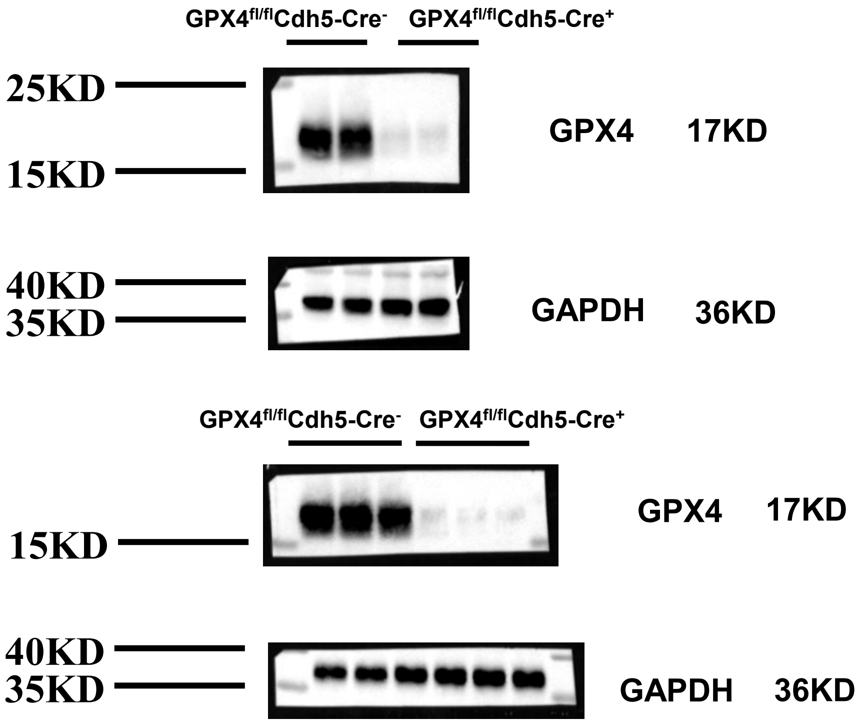

The same membrane were cut to blot for different proteins.

Supplementary Figure 3B

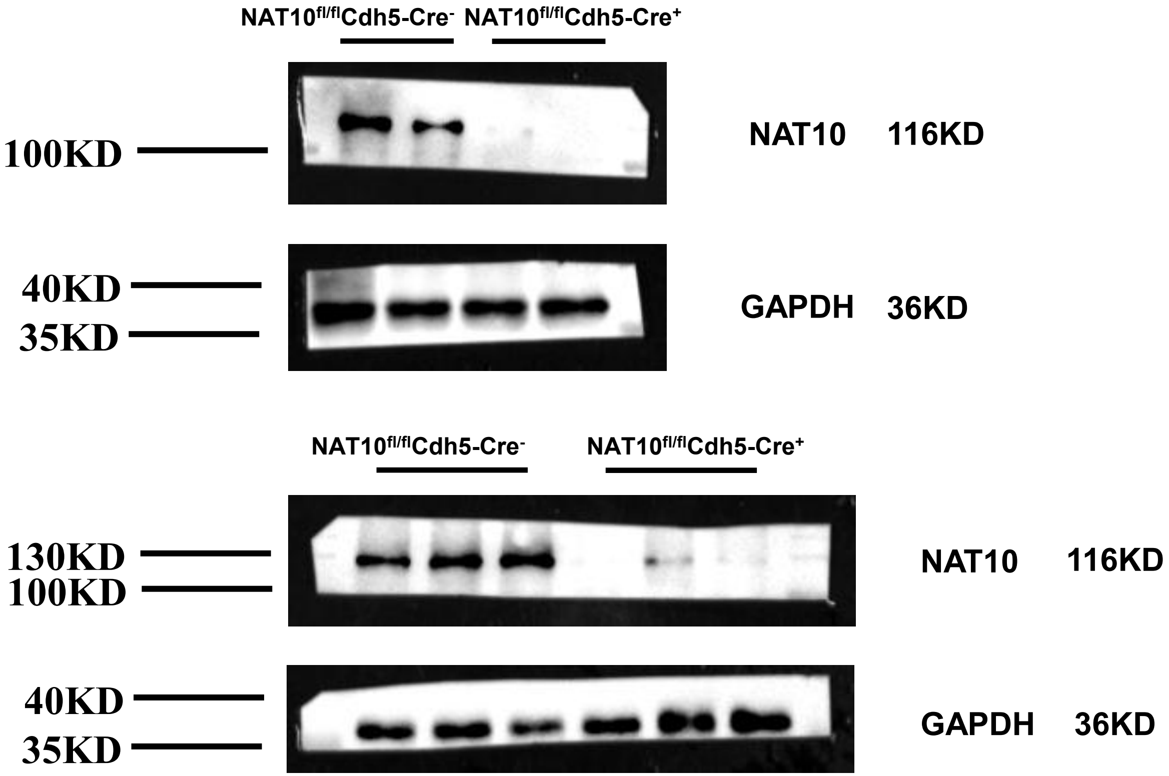

The same membrane were cut to blot for different proteins.

Supplementary Figure 4D

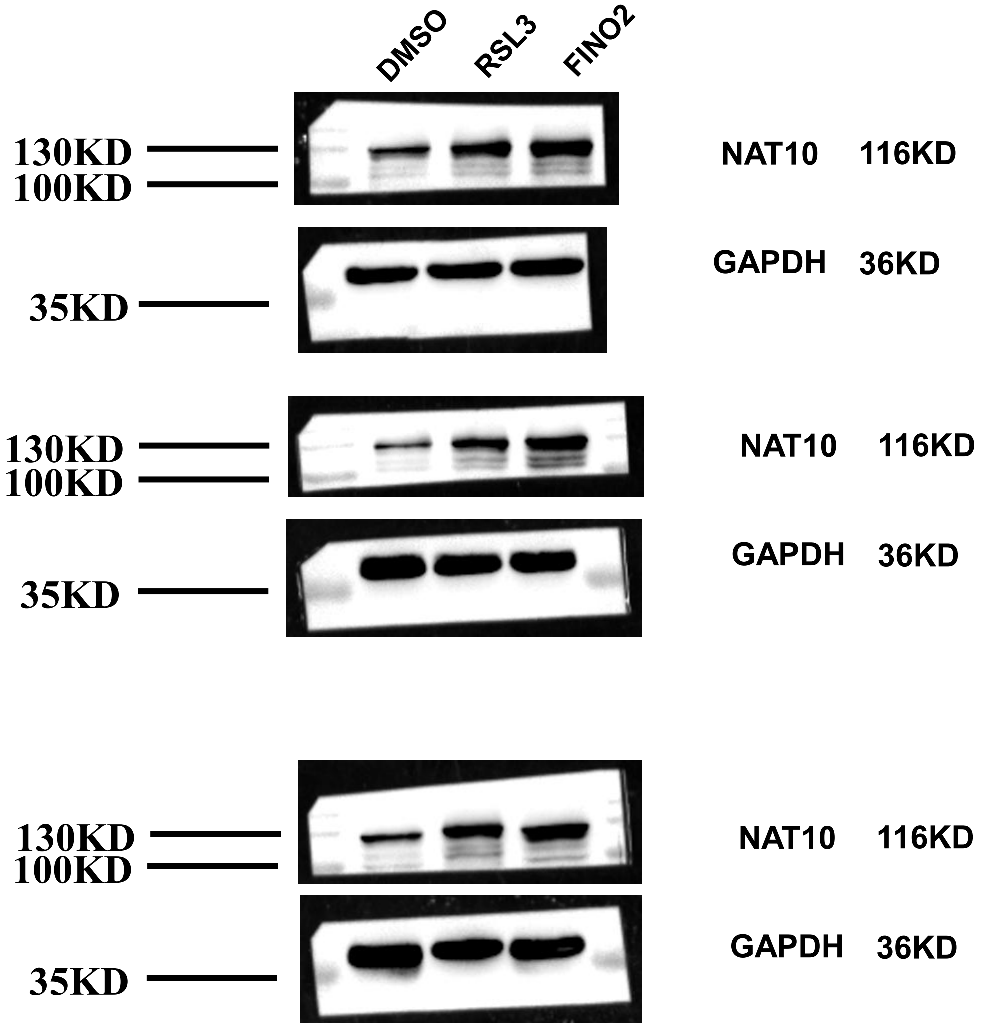

The same membrane were cut to blot for different proteins.

Supplementary Figure 7B

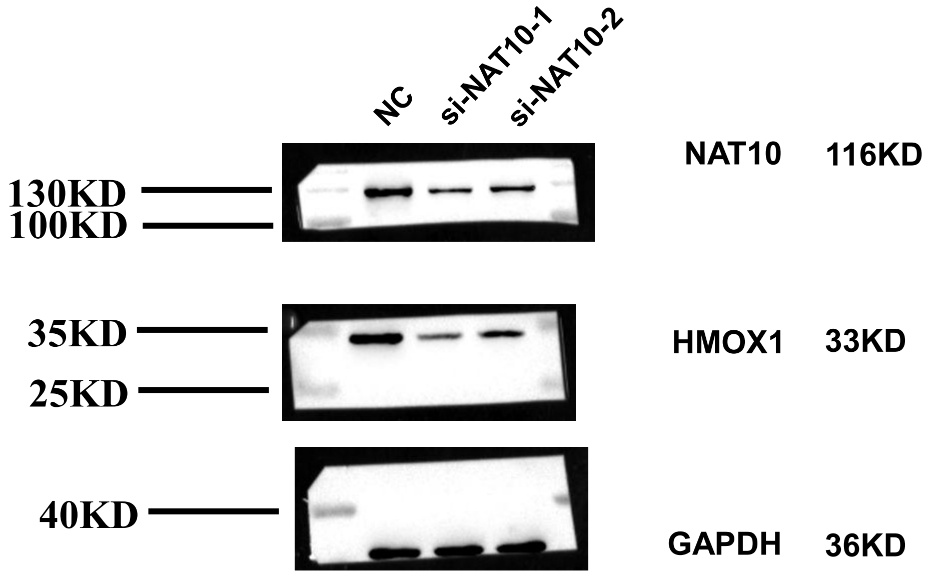

The same membrane were cut to blot for different proteins.

Supplementary Figure 10B

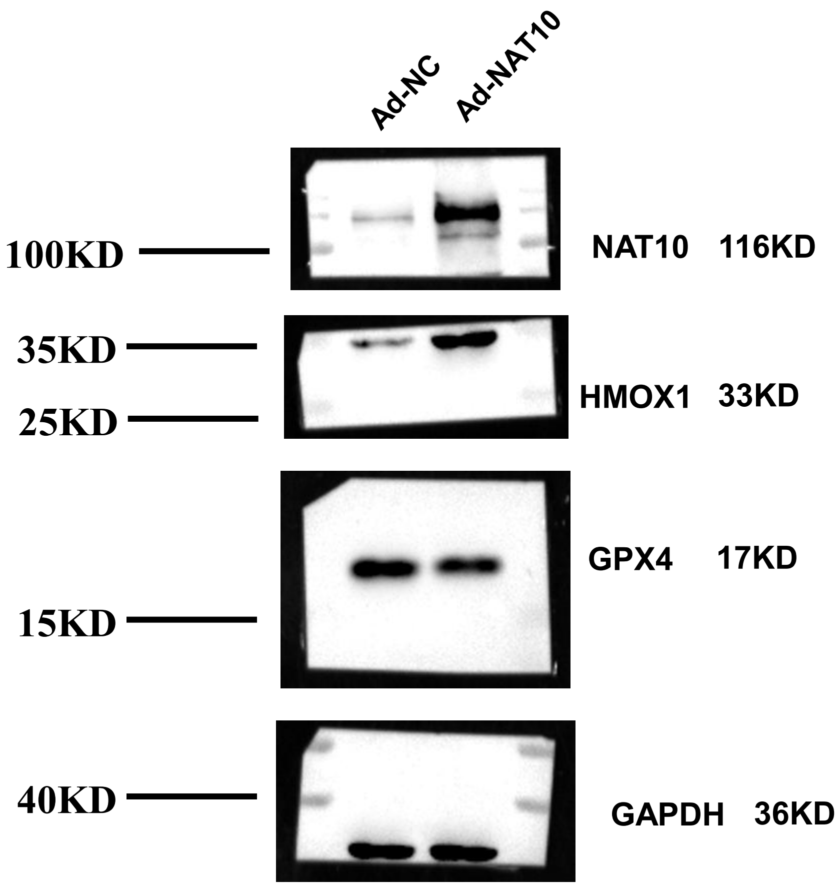

The same membrane were cut to blot for different proteins.

Supplementary Figure 10D

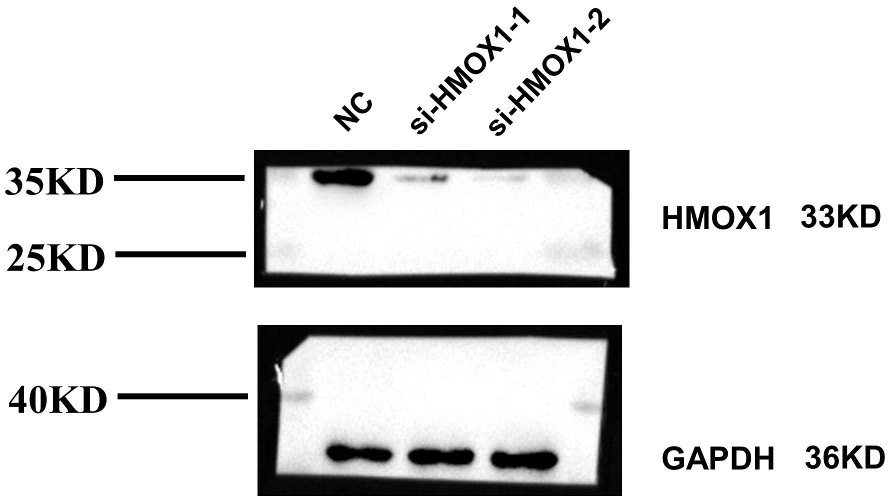

The same membrane were cut to blot for different proteins.

Supplementary Figure 10E

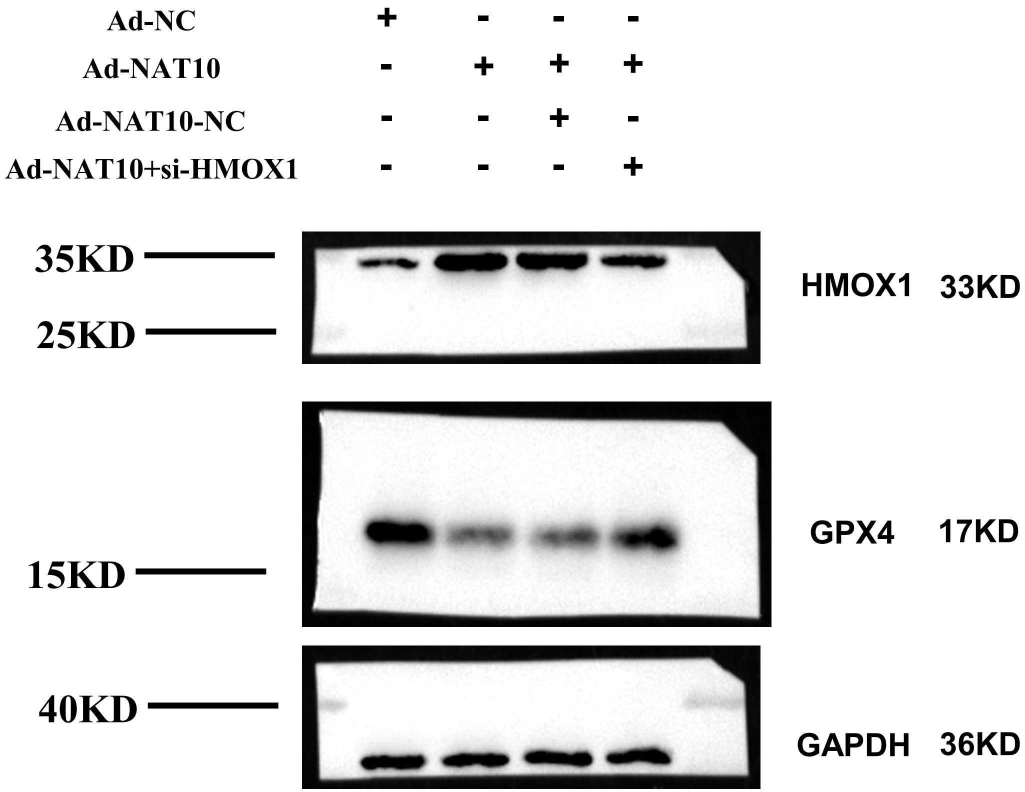

The same membrane were cut to blot for different proteins.

Supplementary Figure 11B

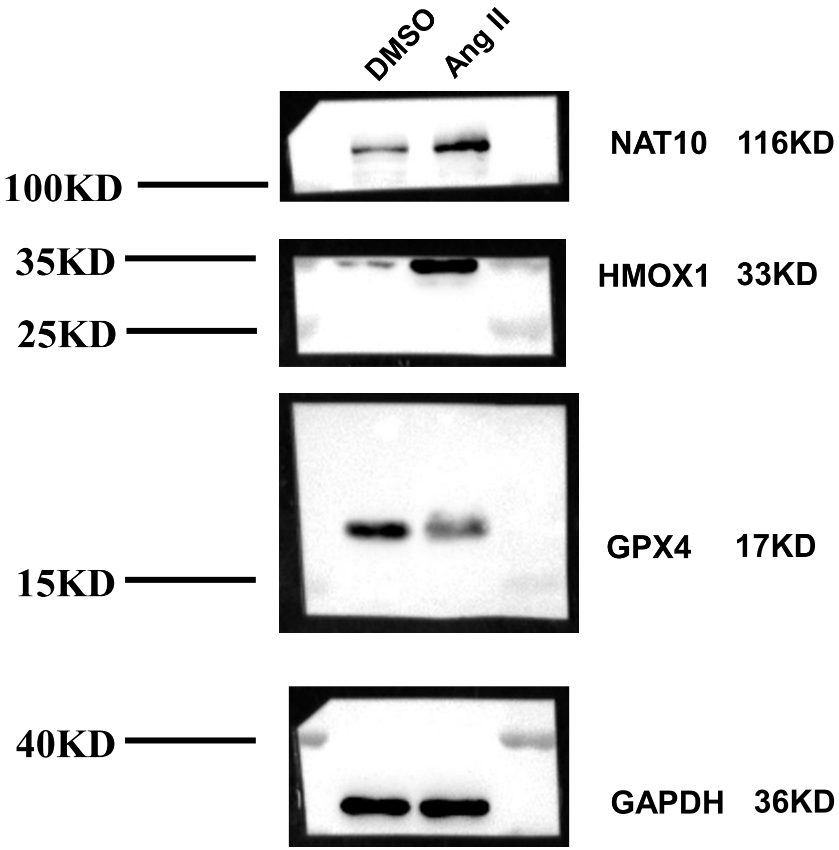

The same membrane were cut to blot for different proteins.

Supplementary Figure 11F

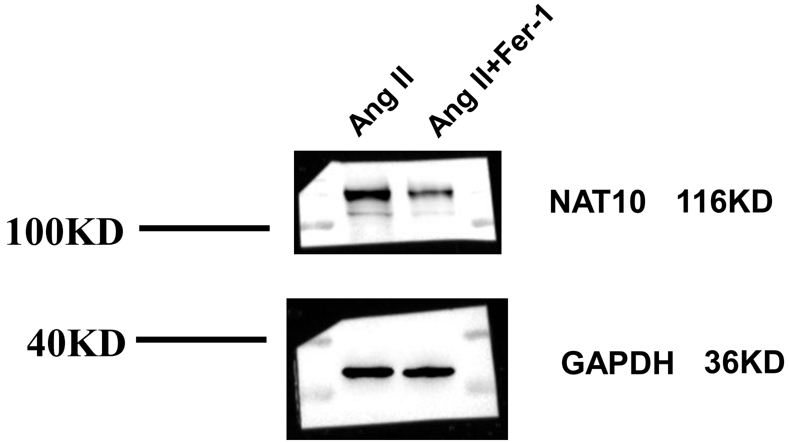

The same membrane were cut to blot for different proteins.

Supplementary Figure 11H

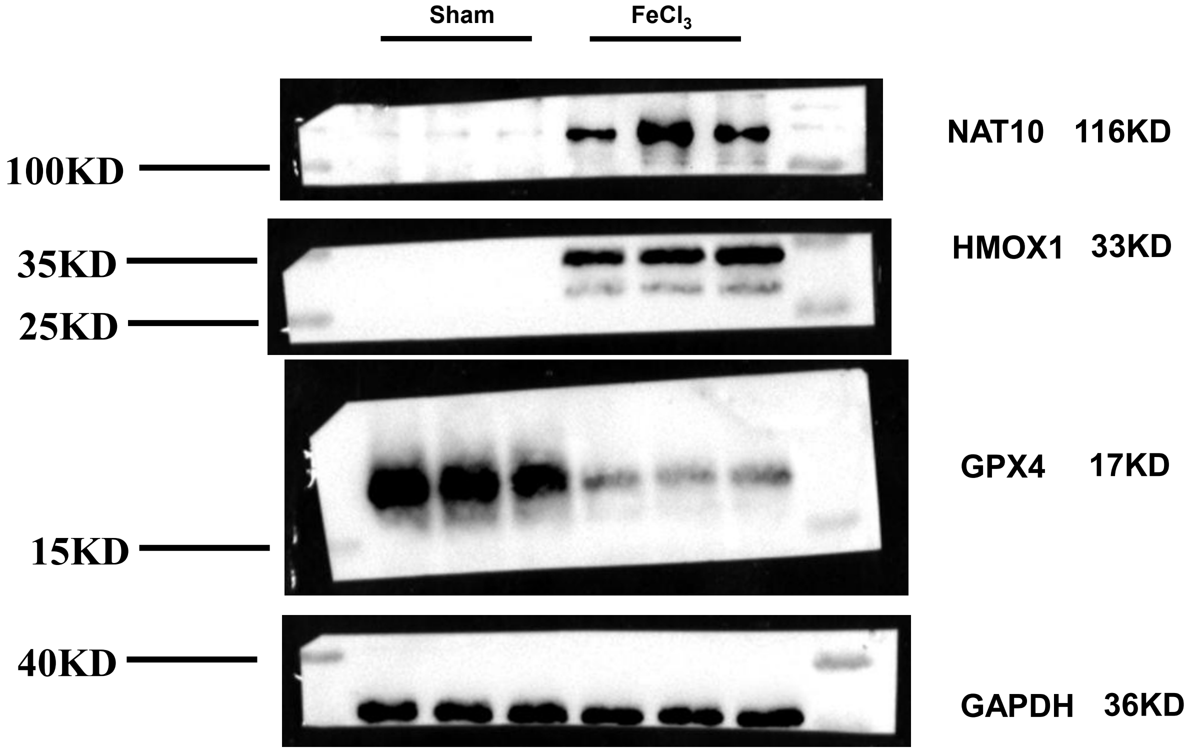

The same membrane were cut to blot for different proteins.
