# Supplementary material for: NAT10-Mediated ac4C-Modification Exacerbates Ferroptosis by Stabilizing HMOX1 in Deep Vein Thrombosis
Source: Arterioscler Thromb Vasc Biol. 2025 Dec 30;46(2):e323986. doi: 10.1161/ATVBAHA.125.323986 (PMC12822780; doi:10.1161/ATVBAHA.125.323986)
Supplement: Supplementary file 2 [file atv-46-e323986-s002.pdf]

SUPPLEMENTAL MATERIALS

NAT10-mediated ac4C-modification exacerbates ferroptosis by stabilizing HMOX1 in Deep vein thrombosis

Yunhong Zhang[a,b,1], Zhen Zhang[a,b,1], Xinkui Liu[a,b], Chu Chu[a,b], Xiaoyan Yu[c], Qiaoqiao Han[c], Wei Li[a,b], Tingting Zhang[a,b], Huiyan Zu[a,b], Nannan Fan[a,b], Ran Wei[d], Feifei Shi[a,b], Fang Li[a,b], Fei Xu[e], Bin Wang[f,*], Xia Li[a,b,g,*]

[a]Innovative Institute of Chinese Medicine and Pharmacy, Shandong University of Traditional Chinese Medicine, Jinan, China;

[b]Key Laboratory of Traditional Chinese Medicine Classical Theory, Ministry of Education, Jinan Key Laboratory of Traditional Chinese Medicine Immunoregulation, Traditional Chinese Medicine Immunoregulation Engineering Research Center of Shandong Province, Shandong University of Traditional Chinese Medicine, Jinan, China;

[c]Shanghai Institute of Immunology, State Key Laboratory of Systems Medicine for Cancer, Shanghai Jiao Tong University School of Medicine, Shanghai, China;

[d]School of Clinical and Basic Medical Sciences, Shandong First Medical University & Shandong Academy of Medical Sciences, Jinan, China;

[e]Department of Respiratory and Critical Care Medicine, Affiliated Hospital of Shandong University of Traditional Chinese Medicine, Jinan, China;

[f]The Second Affiliated Hospital of Shandong University of Traditional Chinese Medicine, Jinan, China;

[g]College of Traditional Chinese Medicine, Shandong University of Traditional Chinese Medicine, Jinan, China;

[1] These authors contributed equally to this work and share first authorship.

[*]Corresponding author:

Bin Wang, The Second Affiliated Hospital of Shandong University of Traditional Chinese Medicine, Jinan 250014, China, Email: 71000848@sdutcm.edu.cn

Xia Li, Innovative Institute of Chinese Medicine and Pharmacy, Shandong University of Traditional Chinese Medicine, 4655 Daxue Road, Changqing District, Jinan 250399, China, Email: 60230033@sdutcm.edu.cn.

Expanded Materials and Methods

Cell treatment

The GV314 (CMV-MCS-3FLAG-SV40-EGFP) adenoviral system (GeneChem, Shanghai, China) was used to obtain NAT10 overexpression adenovirus (Ad-NAT10). HUVECs were cultured at a density of 1×10^5 in a 12-well culture plate for 12 h, then adenovirus (MOI= 200) was added into culture medium for subsequent experiments.

Human NAT10 (NM_024662.3) was obtained from the cDNA library of Genechem (Shanghai, China) with the following primers: NAT10 forward:5'-AGGTCGACTCTAGAGGATCCCGCCACCATGCATCGGAAAAAGGTGGATAACC-3' and reverse: 5'-TCCTTGTAGTCCATACCGGTTTTCTTCCGCTTCAGTTTCATATCTTTTTTG-3'. The adenovirus vector plasmid GV314 (CMV-MCS-3FLAG-SV40-EGFP) (purchased from Shanghai Genechem Co., Ltd.), the vector and NAT10 gene sequence were digested by AgeI and BamHI restriction enzymes, and the complete cloning was achieved through In-fusion recombination method. The sequence of the recombinant vector was verified by DNA sequencing.

DVT mice treatment

We constructed an adeno-associated virus vector to overexpress HMOX1 under the control of the specific TIE promoter in vascular endothelium. Mouse HMOX1 (NM_010442.2) was obtained from the cDNA library of Genechem (Shanghai, China) with the following primers: HMOX1 forward: 5'-GAACCGTCAGATCCGCTAGCCGCCACCATGGAGCGTCCACAGCCCGAC-3' and reverse: 5'-TAGTCCATGGTGGCACCGGTCATGGCATAAATTCCCACTGCCAC-3'. The AAV vector plasmid GV841 (TIE promoter-MCS-3flag-FT2A-EGFP-WPRE-bGH polyA) (purchased from Shanghai Genechem Co., Ltd.), the vector and HMOX1 gene sequence were digested by AgeI and NheI restriction enzymes, and complete cloning was achieved using In-fusion recombination method. The sequence of the recombinant vector was verified by DNA sequencing. 100 ul (5×10^{12} v.g/ml) of adeno-associated virus (AAV-EV or AAV-HMOX1) was injected into NAT10 knockout mice via tail vein.

Supplemental Tables

Table S1. Baseline characteristics of DVT patients and healthy controls.

Characteristics	Control (n=6)	DVT (n=6)
Age, years (mean, SD)	54.8 ± 3.1	57.2 ± 3.2
Sex, females/males	3/3	3/3
Hypertension	0	0
Diabetes mellitus	0	0
Other chronic diseases	0	0

Table S2. The sequences of synthesized siRNAs and negative control (NC).

Gene	Sequences (5'-3')
NC	UUCUCCGAACGUGUCACGUTT
	ACGUGACACGUUCGGAGAATT
si-NAT10-1 (homo sapiens)	CCACCAUCAAUGGCUAUGATT
	UCAUAGCCAUUGAUGGUGGTT
si-NAT10-2 (homo sapiens)	GGCCAAAGCUGUCUUGAAATT
	UUUCAAGACAGCUUUGGCCTT
si-NAT10-3 (homo sapiens)	GCAUGGACCUCUCUGAAUATT
	UAUUCAGAGAGGUCCAUGCTT
si-NAT10-1 (mus musculus)	GGACCAUGAACUCGCUUAATT
	UUAAGCGAGUUCAUGGUCCTT
si-NAT10-2 (mus musculus)	CCCUGUCUCUGAACAUCAUTT
	AUGAUGUUCAGAGACAGGGTT
si-HMOX1-1 (homo sapiens)	CAGAGAAUGCUGAGUUCAUTT
	AUGAACUCAGCAUUCUCUGTT
si-HMOX1-2 (homo sapiens)	GCAACAAAGUGCAAGAUUCTT
	GAAUCUUGCACUUUGUUGCTT

Table S3. Quantitative real-time PCR (qRT-PCR) primers used in this study.

Gene	Sequences (5'-3')
GAPDH (homo sapiens)	F: GCACCGTCAAGGCTGAGAAC

	R: TGGTGAAGACGCCAGTGGA
NAT10 (homo sapiens)	F: GTGGTCTGTCTGGTGGAAGG
	R: CCTGTGGTGTCTCAAGGACC
HMOX1 (homo sapiens)	F: AGTCTTCGCCCCTGTCTACT
	R: CTTCACATAGCGCTGCATGG
GPX4 (homo sapiens)	F: CAGTGAGGCAAGACCGAAGT
	R: CCGAACTGGTTACACGGGAA
ICAM-1 (homo sapiens)	F: CCAGGAGACACTGCAGACAG
	R: CTTCACTGTCACCTCGGTCC
SELP (homo sapiens)	F: TGTTCATCCGCTCACTGCTT
	R: TAGCCTCACAGGTTGGCAAG
TGF-β1 (homo sapiens)	F: TGGTGGAAACCCACAACGAA
	R: GAGCAACACGGGTTCAGGTA
TNF-α (homo sapiens)	F: CCCGAGTGACAAGCCTGTAG
	R: TGAGGTACAGGCCCTCTGAT
eNOS (homo sapiens)	F: TGGCTGGTACATGAGCACTG
	R: GTCTTTCCACAGGGACGAGG
GAPDH (mus musculus)	F: TGTCTCCTGCGACTTCAACA
	R: GGTGGTCCAGGGTTTCTTACT
NAT10 (mus musculus)	F: CGGAAGAAGGTGGATAACCGA
	R: AAAAGAGACCTCTGCCGCTC
HMOX1 (mus musculus)	F: TGACACCTGAGGTCAAGCAC
	R: ATCTTGCACCAGGCTAGCAG
GPX4 (mus musculus)	F: CCATGCACGAATTCTCAGCC
	R: GGTGACGATGCACACGAAAC
ICAM-1 (mus musculus)	F: TTCTCATGCCGCACAGAACT
	R: TCCTGGCCTCGGAGACATTA
SELP (mus musculus)	F: GGGCTTCAGGACAATGGACA
	R: TGGAAGGTGCAGGTTGATCC
TGF-β1 (mus musculus)	F: CTGATACGCCTGAGTGGCTG
	R: TTTGGGGCTGATCCCGTTG
TNF-α (mus musculus)	F: AGCCGATGGGTTGTACCTTG
	R: ATAGCAAATCGGCTGACGGT
eNOS (mus musculus)	F: ATGTCAGGCCCGTACAACAG

	R: CCGCCAAGAGGATACCAGTG
LGMN (mus musculus)	F: GCCTACCAGATCATCCACCG
	R: ACATCTGTGCCGTTAGGTCG
AGPS (mus musculus)	F: TCGCACCTCTTTCTACGTGC
	R: TCAAACACGGTCAGTGGGTC
FAR1 (mus musculus)	F: ATCCTCTCGAACAGGCCTTC
	R: TGGGCTTCTTCCAGTCATCC

Table S4. acRIP-qPCR primers used in this study.

**Gene**	**Sequences (5'-3')**
HMOX1	F: UUCUCCGAACGUGUCACGUTT
	R: ACGUGACACGUUCGGAGAATT

Table S5. The genotypes primers used in this study.

**Gene**	**Sequences (5'-3')**
NAT10	F: CACTTTTCTTCAGTTAGTGCCGAG
	R: TAAAGTCTGGAACCAAAGATCCCA
GPX4	F: AAAGTCCTAGGAAACGCCCG
	R: GGCACTAGGTGGAGGAGTCT
Mutant	F: CACTGGGTCCTGATGGTGCCTATC
	R: TCCTGTTGTTCAGCTTGCACCAG
Wildtype	F: CACTGGGTCCTGATGGTGCCTATC
	R: AATCCAGTCTCTCTTTTGGCGATG
ERT2	F: TTGTGGATTTGACCCTCCATGAT
	R: CACGTTCTTGCACTTCATGCTG

Table S6. Thrombus formation in different groups of DVT mice.

Group	Number of mice	Mice number of thrombus formation	Thrombus formation rate (%)
DVT	120	90	75
DVT+Fer-1	20	11	55
DVT (GPX4$^{fl/fl}$Cdh5-Cre^{-})	20	15	75
DVT (GPX4$^{fl/fl}$Cdh5-Cre^{+})	20	18	90
DVT (NAT10$^{fl/fl}$Cdh5-Cre^{-})	60	45	75
DVT (NAT10$^{fl/fl}$Cdh5-Cre^{+})	40	16	40
DVT+ZnPP	20	11	55
DVT (NAT10$^{fl/fl}$Cdh5-Cre^{+})+CoPP	20	12	60
DVT (NAT10$^{fl/fl}$Cdh5-Cre^{+})+AAV-EV	20	8	40
DVT (NAT10$^{fl/fl}$Cdh5-Cre^{+})+AAV-HMOX1	20	14	70

Supplemental Figures

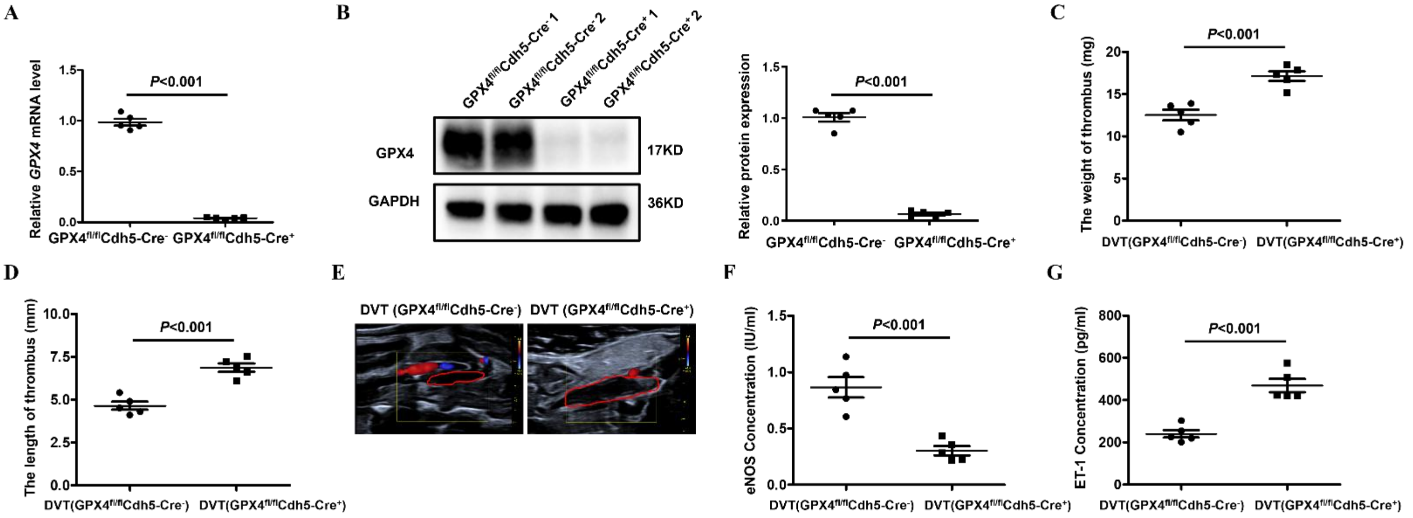

Figure S1. Silencing of GPX4 aggravates the formation of thrombosis in DVT.

(A, B) GPX4 knockdown is detected by qRT-PCR and Western blotting (n= 5 mice per group). (C, D) Thrombus weights and length are measured in the different treatment groups (n= 5 mice per group). (E) Representative images of thrombi in each group detected by vascular ultrasound. (F, G) The plasma levels of eNOS and ET-1 are measured by ELISA (n= 5 mice per group). Results are expressed as mean±SEM. Statistical analysis was performed by Welch's *t*-test (A, B) and two-tailed Student *t* test (C, D, F, G).

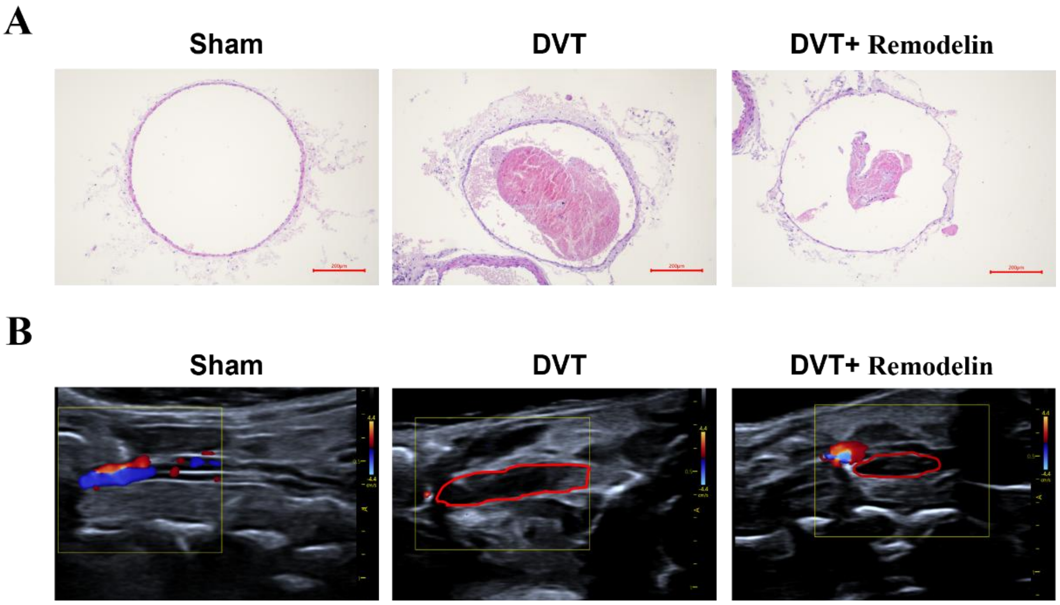

Figure S2. Targeting NAT10 with Remodelin alleviates the formation of thrombosis.

(A, B) Representative images of thrombi detected by H&E staining (magnification, ×100) and vascular ultrasound by treating with Remodelin (NAT10 inhibitor). Scale bars= 200 μm.

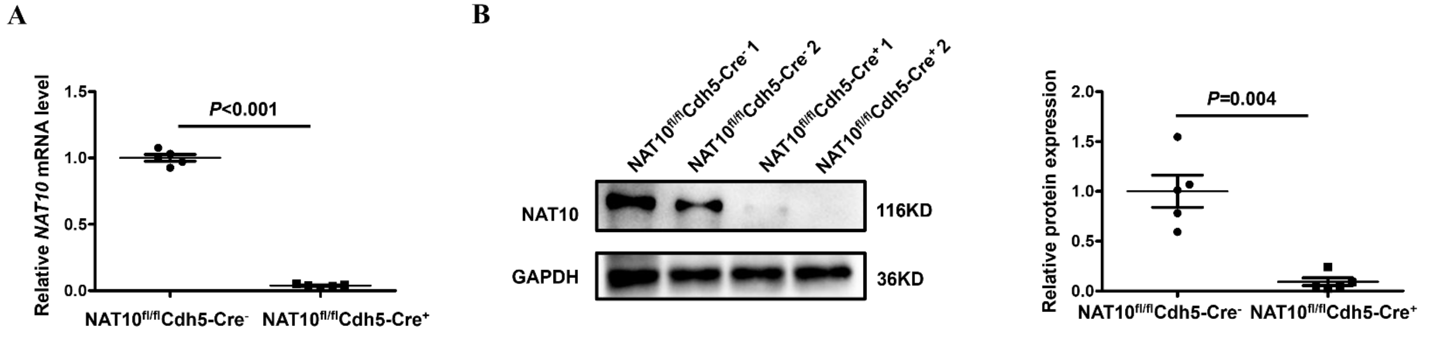

Figure S3. The expression of NAT10 in NAT10 knockout mice.

(A) Knockdown of NAT10 mRNA levels was detected by qRT-PCR analysis (n= 5 mice per group). (B) Knockdown of NAT10 protein levels was detected by Western blotting (n= 5 mice per group). Results are expressed as mean±SEM. Statistical analysis was performed by Welch's *t*-test (A, B).

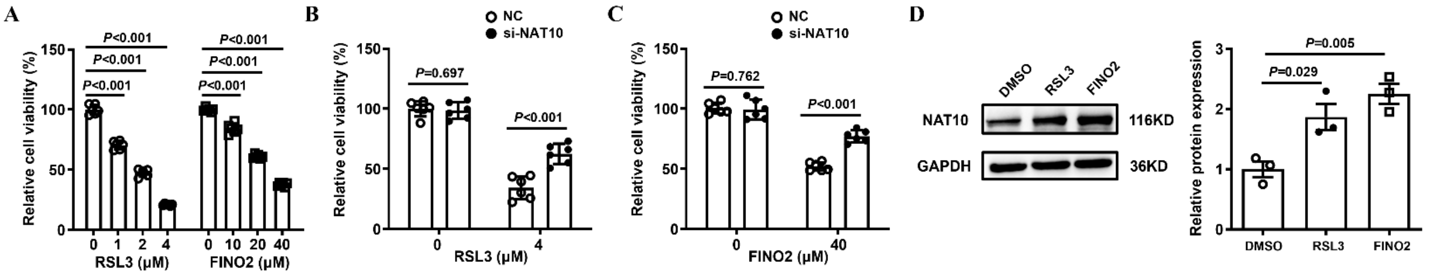

Figure S4. Inhibition of NAT10 attenuated ferroptosis in HUVECs.

(A) The concentration of RSL3 and FINO2 induced ferroptosis (n= 6). (B, C) NAT10 knockdown improved cell viability as shown by CCK8 assay in ferroptosis (n= 6). (D) The protein levels of NAT10 after induction of ferroptosis were measured by Western blotting (n= 3). Results are expressed as mean±SEM. The "n" refers to the number of biological replicates. Statistical analysis was performed by two-tailed Student *t* test (B, C) and one-way ANOVA with Tukey post hoc tests (A, D).

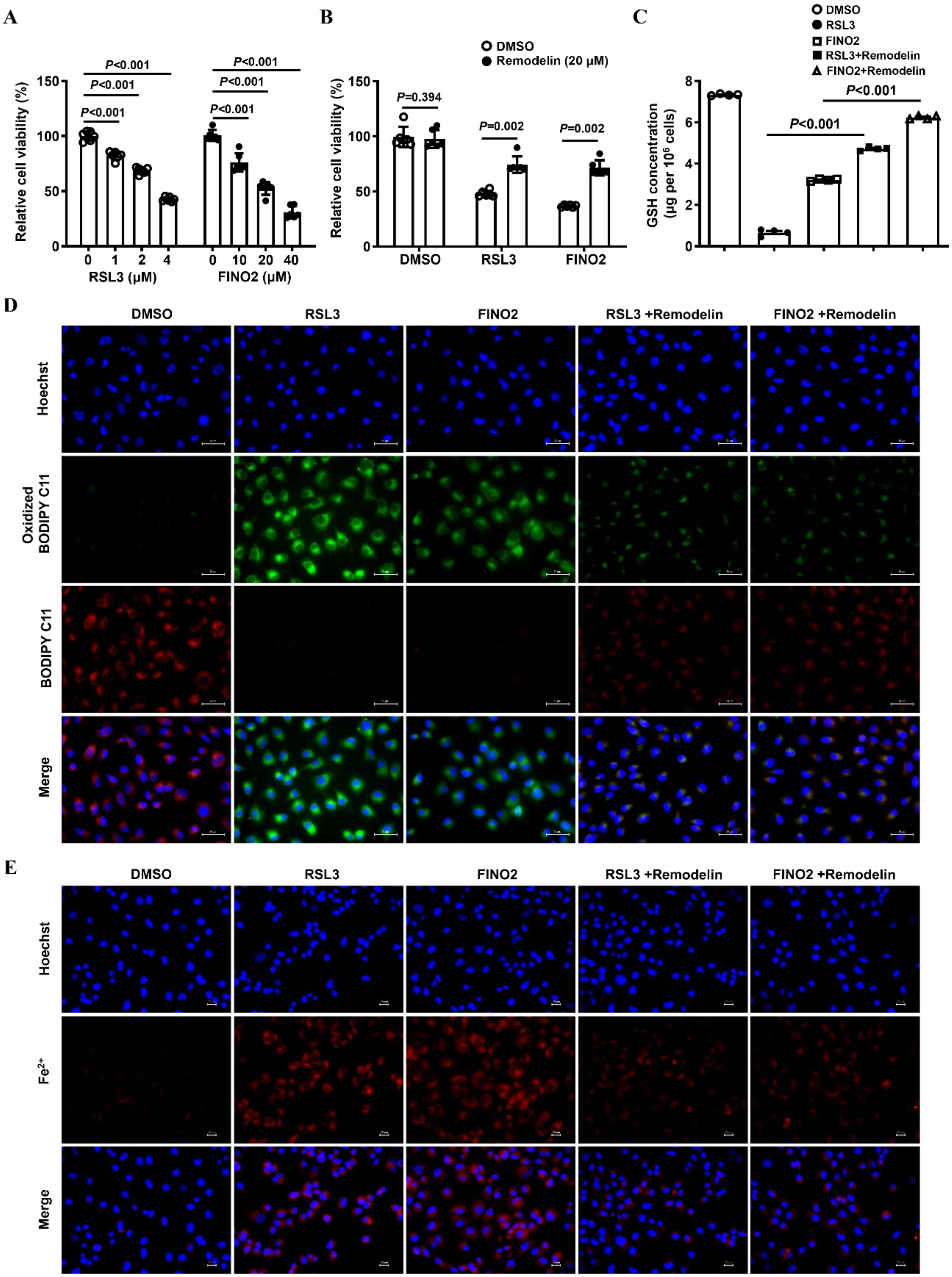

Figure S5. Knockdown of NAT10 inhibits ferroptosis in C166 cells.

(A) The concentration of RSL3 and FINO2 induced ferroptosis (n= 6). (B) Inhibition of NAT10 detected cell viability in ferroptosis (n= 6). (C) The levels of GSH detected in C166 cells (n= 4). (D) Lipid peroxidation detected by BODIPYTM 581/591 C11 probe inhibit NAT10 followed by RSL3 (4 μM) or FINO2 (40 μM) treatment for 12 h in C166 cells (magnification, ×400). Green images represent oxidized lipids, while red images represent non-oxidized lipids. Scale bars= 50 μm. (E) FerroOrange probes showed Fe^{2+} after inhibit NAT10

followed by RSL3 or FINO2 treatment for 12 h in C166 cells (magnification, ×400). Red in images represents Fe^{2+}; blue represents the nucleus. Scale bars= 20 μm. Results are expressed as mean±SEM. The "n" refers to the number of biological replicates. Statistical analysis was performed by one-way ANOVA with Tukey post hoc tests (A, C) and Mann-Whitney U test (B).

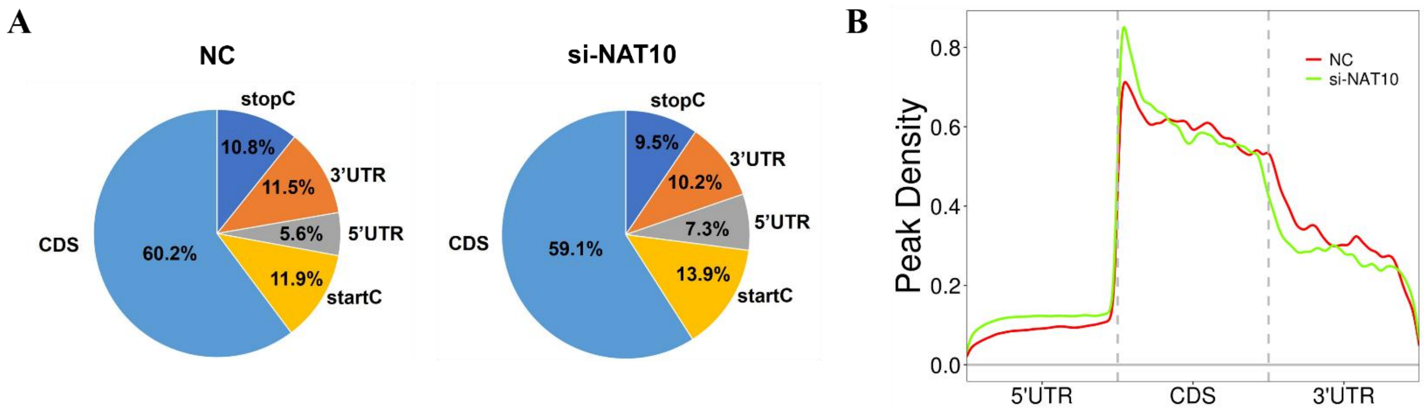

Figure S6. The sequential analysis of ac4C modifications.

(A) Proportion of ac4C peak distribution region across the entire set of mRNA transcripts. (B) Density distribution of ac4C peaks across mRNA transcripts.

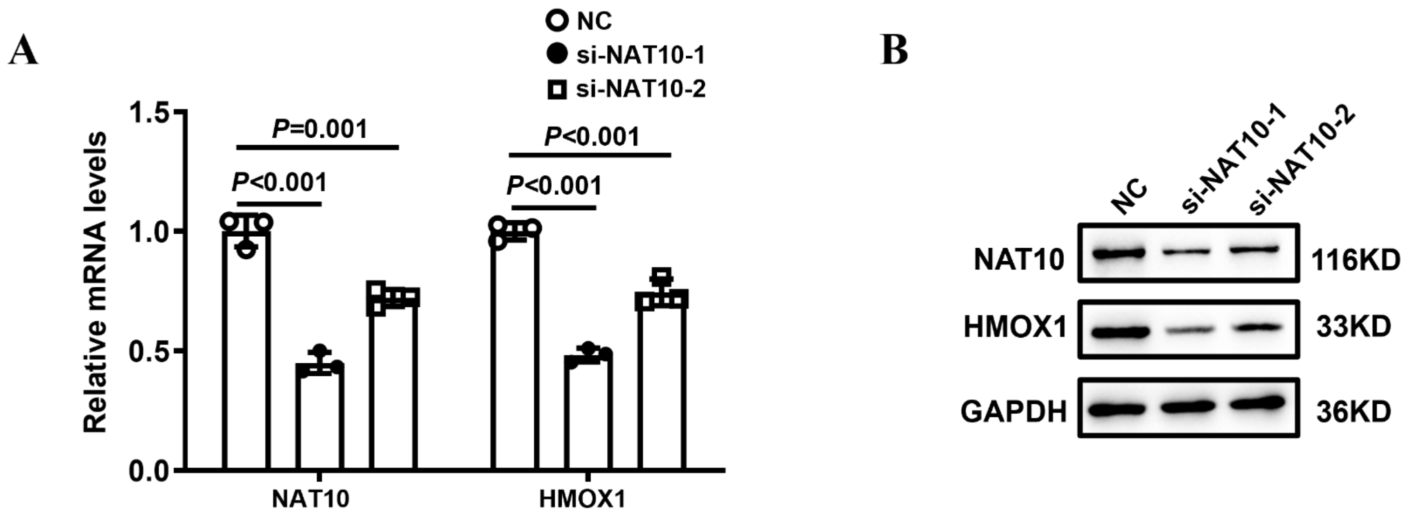

Figure S7. Inhibition of NAT10 expression in C166 cells.

(A) Inhibition of NAT10 mRNA levels was detected by qRT-PCR (n= 3). (B) Inhibition of NAT10 protein levels was detected by Western blotting. Results are expressed as mean±SEM. The "n" refers to the number of biological replicates. Statistical analysis was performed using the one-way ANOVA with Tukey correction for A.

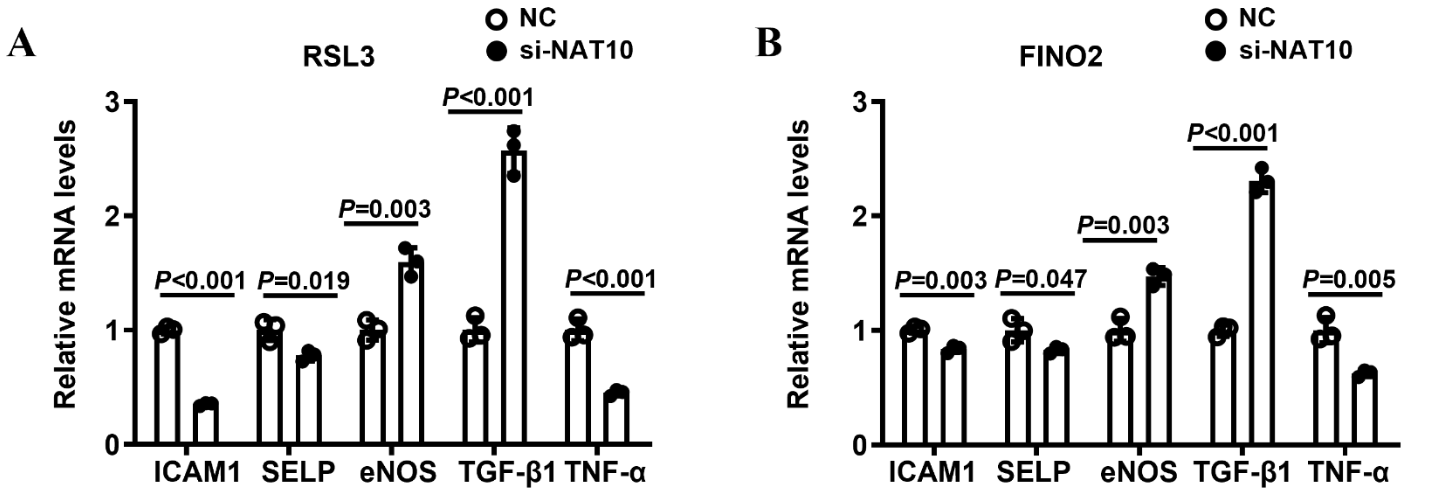

Figure S8. NAT10 downregulation ameliorates endothelial dysfunction in HUVECs.

(A, B) Expressions of ICAM1, SELP, eNOS, TGF-β1 and TNF-α are determined by qRT-PCR in different treatment groups (n= 3). Results are expressed as mean ± SEM. The "n" refers to the number of biological replicates. Statistical analysis was performed by two-tailed Student *t* test (A, B).

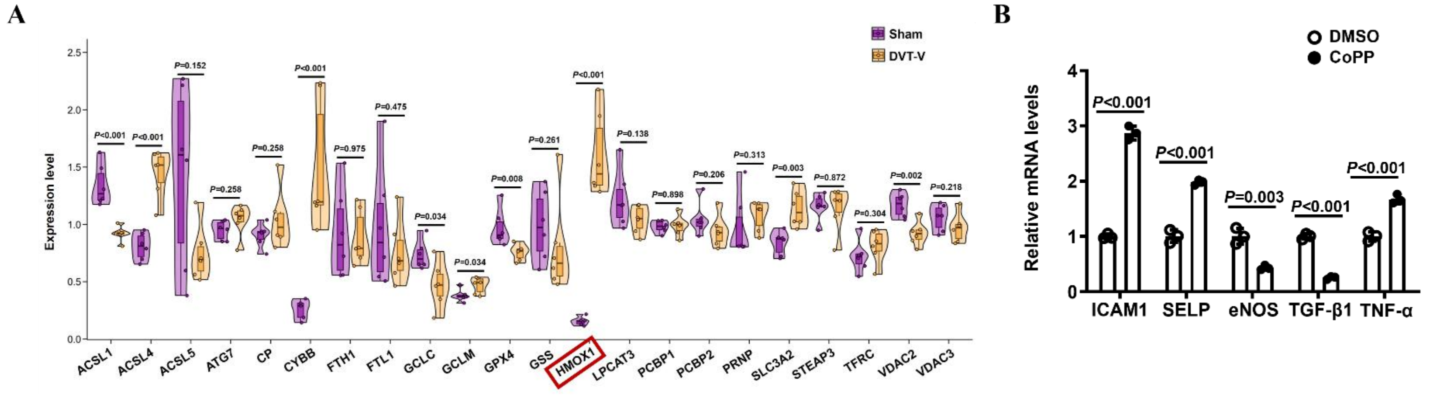

Figure S9. HMOX1 promotes the formation of DVT.

(A) The expression of HMOX1 in 4D Label Free of DVT mice (n= 6 mice per group). (B) Expressions of ICAM1, SELP, eNOS, TGF-β1 and TNF-α were determined by qRT-PCR (n= 3 mice per group). Results are expressed as mean±SEM. Statistical analysis was performed by two-tailed Student *t* test (A, B).

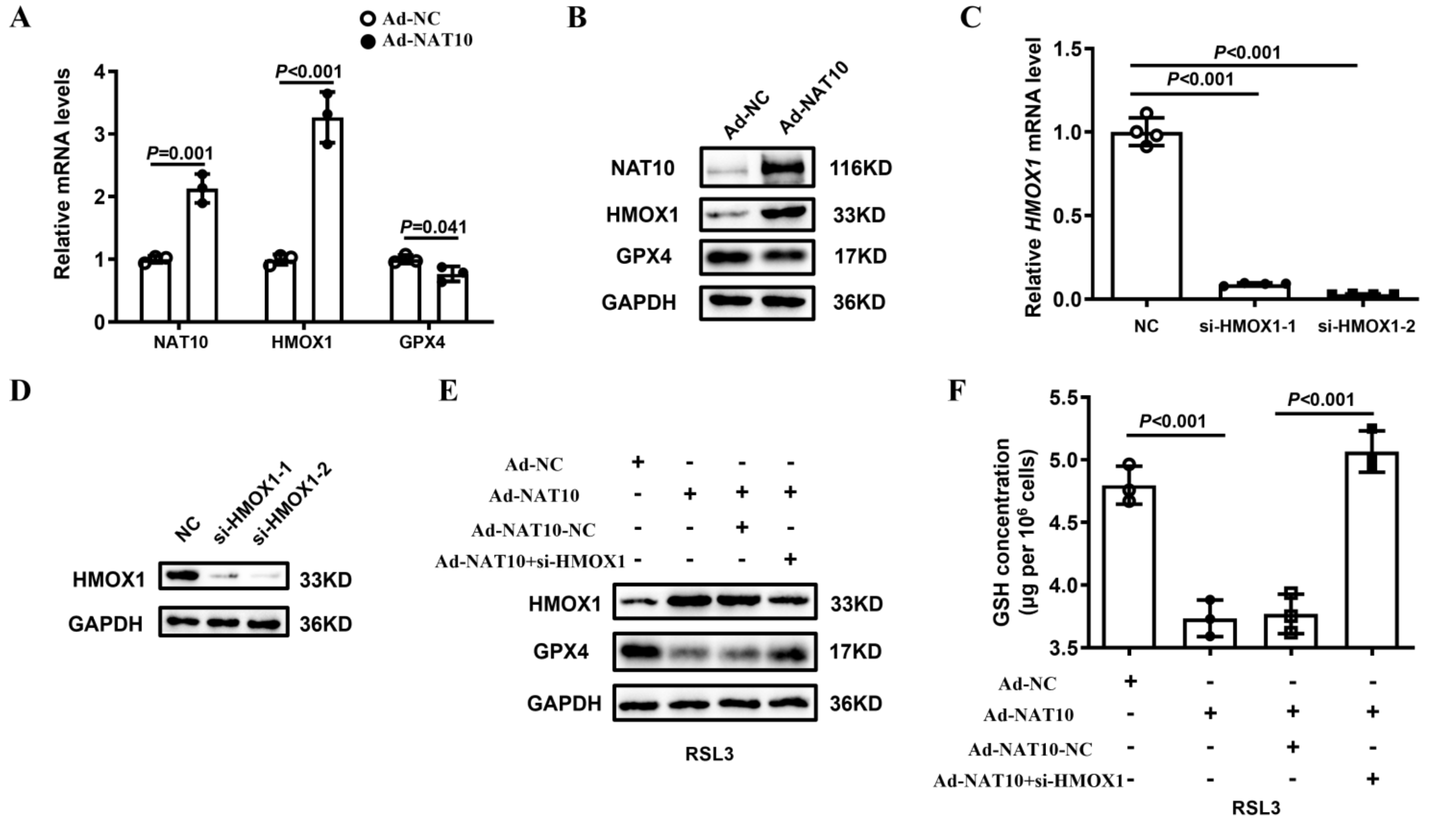

Figure S10. Overexpression of NAT10 exacerbate HMOX1 in HUVECs.

(A, B) The mRNA and protein levels of NAT10, HMOX1, and GPX4 were detected by overexpression of NAT10. (C, D) The HMOX1 mRNA and protein levels are detected by inhibition of HMOX1. (E) The protein levels of HMOX1 and GPX4 in each treatment group were measured in RSL3-induced ferroptosis. (F) The levels of GSH were measured in RSL3-induced ferroptosis. Results are expressed as mean ± SEM. Statistical analysis was performed by two-tailed Student *t* test (A) and one-way ANOVA with Tukey post hoc tests (C, F).

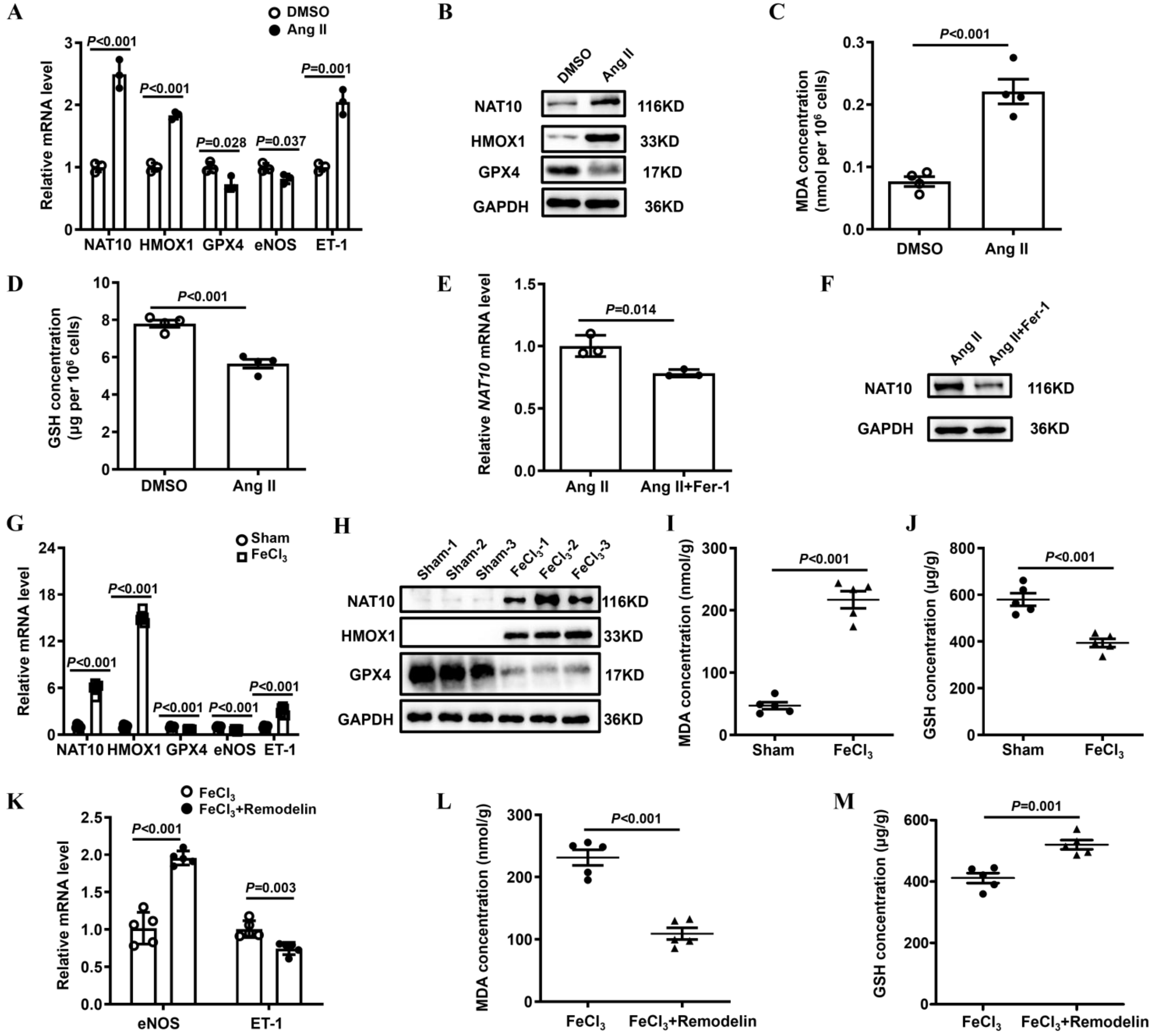

Figure S11. Inhibition of NAT10 alleviates endothelial injury and ferroptosis.

(A) The mRNA levels of NAT10, HMOX1, GPX4, eNOS, and ET-1 were detected by qRT-PCR in HUVECs treated with Ang II (100 μM) for 24 h (n= 3). (B) The protein levels of NAT10, HMOX1 and GPX4 were detected by Western blotting in HUVECs treated with Ang II (100 μM) for 24 h. (C, D) The levels of MDA and GSH were detected in HUVECs after treatment (n= 4). (E, F) The mRNA and protein levels of NAT10 were detected after treating the effects of ferroptosis inhibition (Fer-1, 10 μM) on Ang II-induced endothelial injury. (G) The mRNA levels of NAT10, HMOX1, GPX4, eNOS, and ET-1 were detected by qRT-PCR in 10% Ferric-chloride ($FeCl_3$)-induced mice (n= 6 mice per group). (H) The protein levels of NAT10, HMOX1, and GPX4 were detected by Western blotting in $FeCl_3$-induced mice. (I, J, L, M) The levels of GSH and MDA were detected in mice after treatment (n= 5 mice per group). (K) The mRNA levels of eNOS and ET-1 were measured in $FeCl_3$-induced mice

following treatment with the NAT10 inhibitor (Remodelin) (n= 5 mice per group). Results are expressed as mean ±SEM. The "n" refers to the number of biological replicates. All experiments were independently repeated at least three times. Results are expressed as mean±SEM. Statistical analysis was performed by two-tailed Student *t* test (A, C-E, G, I-M).

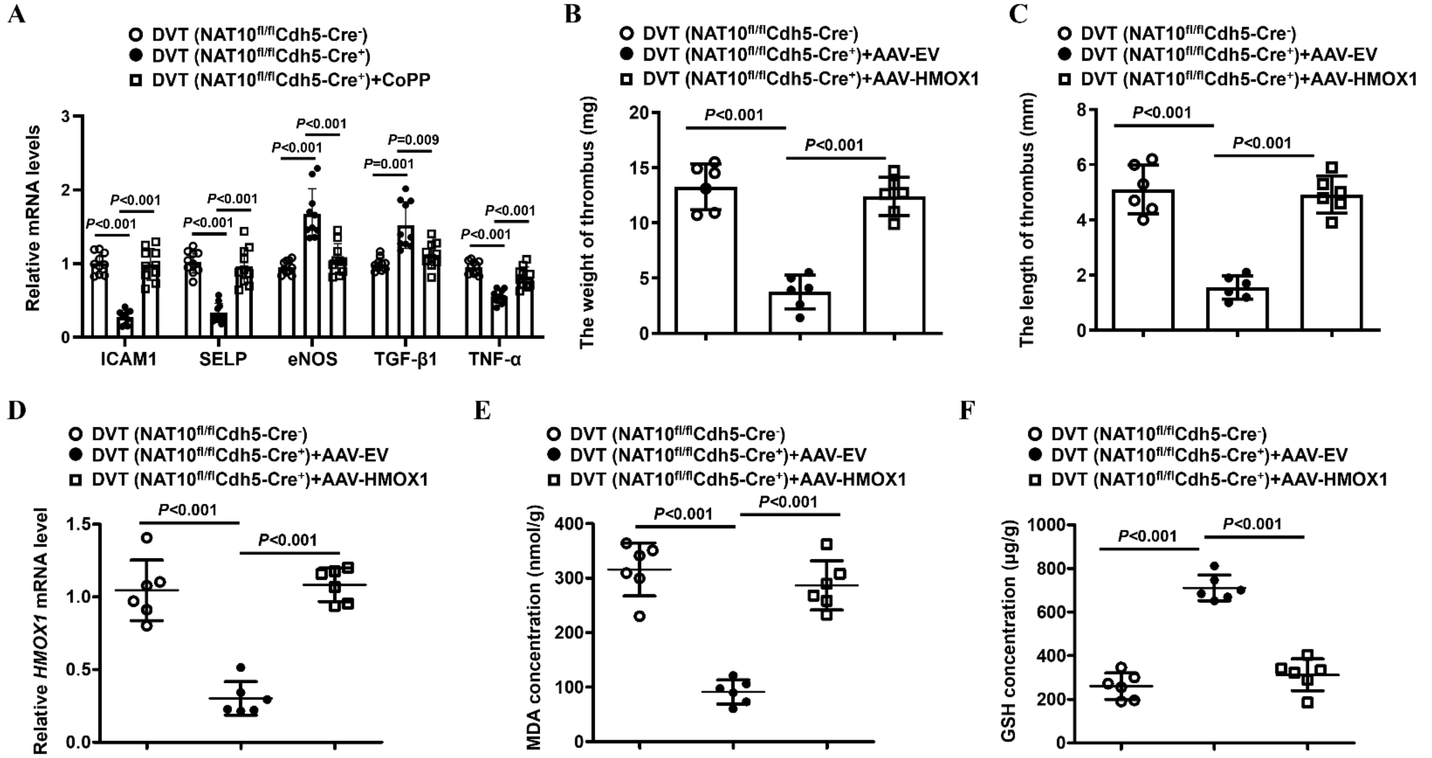

Figure S12. Overexpression of HMOX1 promotes thrombosis in NAT10-knockout DVT mice.

(A) Expressions of ICAM1, SELP, eNOS, TGF-β1 and TNF-α are determined by qRT-PCR in different treatment groups (n= 10 mice per group). (B, C) Thrombus weights and length are measured in the different treatment groups (n= 6 mice per group). (D) The expression of HMOX1 is determined by qRT-PCR in different treatment groups (n= 6 mice per group). (E, F) Relative expression levels of MDA and GSH in different treatment groups (n= 6 mice per group). Results are expressed as mean±SEM. Statistical analysis was performed by one-way ANOVA with Tukey post hoc tests (ICAM-1, SELP, and TNF-α of A, B-F) and Brown-Forsythe ANOVA with Games-Howell post hoc tests (eNOS and TGF-β1 of A).

Major Resources Table

In order to allow validation and replication of experiments, all essential research materials listed in the Methods should be included in the Major Resources Table below. Authors are encouraged to use public repositories for protocols, data, code, and other materials and provide persistent identifiers and/or links to repositories when available. Authors may add or delete rows as needed.

Animals (in vivo studies)

Species	Vendor or Source	Background Strain	Sex	Persistent ID / URL
Mouse	Beijing HFK Bioscience Company	C57BL/6J	Male	http://www.hfkbio.com/cms/item/view?table=product&id=60

Genetically Modified Animals

	Species	Vendor or Source	Background Strain	Other Information	Persistent ID / URL
**Parent - Male and Female**	Mouse	Cyagen Biosciences	C57BL/6J	$NAT10^{flox/flox}$	https://www.cyagen.cn/mice-bank/S-CKO-17344
**Parent - Male and Female**	Mouse	Cyagen Biosciences	C57BL/6J	Cdh5-Cre^{ERT2}	Custom-made
**Parent - Male and Female**	Mouse	Shanghai Jiao Tong University (SHANGHAI MODEL ORGANISMS)	C57BL/6J	$GPX4^{flox/flox}$	https://www.modelorg.com/portal/article/index/id/9927/post_type/3.html

Antibodies

Target antigen	Vendor or Source	Catalog #	Working concentration	Lot # (preferred but not required)	Persistent ID / URL
NAT10	Abcam	ab194297	0.606 ug/ml	1002492-12	https://www.abcam.cn/products/primary-antibodies/nat10-antibody-epr18663-ab194297.html
HMOX1	Abcam	ab68477	0.043 ug/ml	1000692-6	https://www.abcam.cn/products/primary-antibodies/heme-oxygenase-1-antibody-epr1390y-ab68477.html
GPX4	Abcam	ab125066	0.485 ug/ml	1000287-36	https://www.abcam.cn/products/primary-antibodies/glutathione-peroxidase-4-antibody-epncir144-ab125066.html
GAPDH	Abcam	ab181603	0.1485 ug/ml	1016808-1	https://www.abcam.cn/products/primary-antibodies/gapdh-antibody-epr16884-loading-control-ab181603.html

Anti-N4-acetylcytidine (ac4C) antibody	Abcam	ab252215	1.222 ug/ml	1026348-3	https://www.abcam.cn/products/primary-antibodies/n4-acetylcytidine-ac4c-antibody-eprnci-184-128-ab252215.html
Horseradish enzyme-conjugated goat anti-rabbit IgG (H+L)	ZSGB-BIO	ZB-2301	0.1 ug/ml	226731013	http://www.zsbio.com/product/ZB-2301

DNA/cDNA Clones

Clone Name	Sequence	Source / Repository	Persistent ID / URL
None			

Cultured Cells

Name	Vendor or Source	Sex (F, M, or unknown)	Persistent ID / URL
human umbilical vein endothelial cells	BeNa Culture Collection	unknown	https://www.bncc.com/pro/p1/1/p_378266.html
C166	BeNa Culture Collection	unknown	https://www.bncc.com/pro/p1/1/p_317467.html

Data & Code Availability

Description	Source / Repository	Persistent ID / URL
None		

Other

Description	Source / Repository	Persistent ID / URL
RSL3	aladdin	https://www.aladdin-e.com/zh_cn/r302648.html
FINO2	Selleck	https://www.selleck.cn/products/fino2.html
Ferrostatin-1	aladdin	https://www.aladdin-e.com/zh_cn/f129882.html
CoPP	aladdin	https://www.aladdin-e.com/zh_cn/c115571.html
ZnPP	Macklin	https://www.macklin.cn/search/Z836208
actinomycin D	AbMole	https://www.abmole.cn/products/actinomycin-d.html?authstamp=9d04051f9375f56618b255cd5c685269
Remodelin	aladdin	https://www.aladdin-e.com/zh_cn/r288492.html

ARRIVE GUIDELINES

The ARRIVE guidelines (https://arriveguidelines.org/) are a checklist of recommendations to improve the reporting of research involving animals. Key elements of the study design should be included below to better enable readers to scrutinize the research adequately, evaluate its methodological rigor, and reproduce the methods or findings.

Study Design

Groups	Sex	Age (weeks)	Number (prior to experiment)	Number (after termination)	Littermates (Yes/No)	Other description
Sham	male	8	80	80	Unknown	

DVT	male	8	120	90	Unknown	
DVT+Fer-1	male	8	20	11	Yes	
DVT (GPX4$^{fl/fl}$ Cdh5-Cre^{-})	male	8	20	15	Yes	
DVT (GPX4$^{fl/fl}$Cdh5-Cre^{+})	male	8	20	18	Yes	
DVT (NAT10$^{fl/fl}$ Cdh5-Cre^{-})	male	8	60	45	Yes	
DVT (NAT10$^{fl/fl}$Cdh5-Cre^{+})	male	8	40	16	Yes	
DVT+ZnPP	male	8	20	11	Yes	
DVT (NAT10$^{fl/fl}$Cdh5-Cre^{+})+CoPP	male	8	20	12	Yes	
DVT (NAT10$^{fl/fl}$Cdh5-Cre^{+})+AAV-EV	male	8	20	8	Yes	
DVT (NAT10$^{fl/fl}$Cdh5-Cre^{+})+AAV-HMOX1	male	8	20	14	Yes	

Sample Size: Please explain how the sample size was decided Please provide details of any a *prior* sample size calculation, if done.

No statistical method was used to predetermine sample size.

Inclusion Criteria

All mice with appropriate genotypes and age were included in the experiments.

Exclusion Criteria

Murine Doppler ultrasound examination excluded mice without obvious blood clots from the DVT group.

Randomization

A randomization process was performed in grouping mice with the same phenotypes.

Blinding

Animal treatments and histological analyses were performed in a single-blinded fashion. No blinding was used for the remaining analyses.
